# E3 ligase TRIM28 promotes anti-PD-1 resistance in non-small cell lung cancer by enhancing the recruitment of myeloid-derived suppressor cells

**DOI:** 10.1186/s13046-023-02862-3

**Published:** 2023-10-21

**Authors:** Manman Liang, Zhengui Sun, Xingwu Chen, Lijing Wang, Hanli Wang, Lilong Qin, Wenying Zhao, Biao Geng

**Affiliations:** 1 Department of Internal Medicine, Yijishan Hospital, The First Affiliated Hospital of Wannan Medical College, Wuhu, 241000 Anhui China; 2 Department of Respiratory Medicine, Yijishan Hospital, The First Affiliated Hospital of Wannan Medical College, 2 Zheshan West Road, Wuhu, 241000 Anhui China; 3 Department of Medical Oncology, Yijishan Hospital, The First Affiliated Hospital of Wannan Medical College, Wuhu, 241000 Anhui China

**Keywords:** NSCLC, TRIM28, NF-κB signaling, MDSCs, Tumor microenvironment

## Abstract

**Background:**

Alterations in several tripartite motif-containing (TRIM) family proteins have been implicated in the pathogenesis of lung cancer. TRIM28, a member of the TRIM E3 ligase family, has been associated with tumorigenesis, cell proliferation, and inflammation. However, little is known about TRIM28 expression and its role in the immune microenvironment of non-small cell lung cancer (NSCLC).

**Methods:**

We assessed the clinical significance of TRIM28 in tissue microarrays and TCGA cohorts. We investigated the function of TRIM28 in syngeneic mouse tumor models, the *Kras*^*LSL−G12D/+*^; *Tp53*^*fl/fl*^ (KP) mouse model, and humanized mice. Immune cell composition was analyzed using flow cytometry and immunohistochemistry.

**Results:**

Our findings revealed a positive correlation between TRIM28 expression and the infiltration of suppressive myeloid-derived suppressor cells (MDSCs) in NSCLC. Moreover, silencing TRIM28 enhanced the efficacy of anti-PD-1 immunotherapy by reshaping the inflamed tumor microenvironment. Mechanistically, we demonstrated that TRIM28 could physically interact with receptor-interacting protein kinase 1 (RIPK1) and promote K63-linked ubiquitination of RIPK1, which is crucial for sustaining activation of the NF-κB pathway. Mutagenesis of the E3 ligase domain corroborated the essential role of E3 ligase activity in TRIM28-mediated NF-κB activation. Further experiments revealed that TRIM28 could upregulate the expression of CXCL1 by activating NF-κB signaling. CXCL1 could bind to CXCR2 on MDSCs and promote their migration to the tumor microenvironment. TRIM28 knockdown increased responsiveness to anti-PD-1 therapy in immunocompetent mice, characterized by increased CD8^+^T tumor-infiltrating lymphocytes and decreased MDSCs.

**Conclusion:**

The present study identified TRIM28 as a promoter of chemokine-driven recruitment of MDSCs through RIPK1-mediated NF-κB activation, leading to the suppression of infiltrating activated CD8^+^T cells and the development of anti-PD-1 resistance. Understanding the regulation of MDSC recruitment and function by TRIM28 provides crucial insights into the association between TRIM28 signaling and the development of an immunosuppressive tumor microenvironment. These insights may inform the development of combination therapies to enhance the effectiveness of immune checkpoint blockade therapy in NSCLC.

**Supplementary Information:**

The online version contains supplementary material available at 10.1186/s13046-023-02862-3.

## Introduction

Lung cancer is reportedly the leading cause of cancer-related fatalities worldwide, primarily due to its often advanced stage at diagnosis, metastatic potential, and resistance to therapeutic interventions [1]. A hallmark of cancer is its capacity to evade immune surveillance through various mechanisms, including the dysregulation of immune checkpoint pathways [2, 3]. While cancer immunotherapy has shown promise in improving progression-free and overall survival (OS), challenges such as drug resistance and adverse reactions remain major obstacles in its management [4]. Consequently, there exists an urgent need to gain a deeper understanding of the cellular and molecular mechanisms governing the antitumor immune response. This knowledge can help identify novel therapeutic targets and develop predictive biomarkers for the response to immune-based therapies [5].

Recent evidence suggests that genetic aberrations within cancer cells substantially influence the immune composition of tumors [6, 7]. It is now understood that specific cancer-associated genes, in addition to driving intrinsic cancer cell programs, also alter the secretome of cancer cells, thereby reshaping the immune microenvironment [8]. Clinical studies increasingly indicate that genetic aberrations in human cancers correlate with changes in immune composition and responses to immunotherapy [9]. Tripartite motif (TRIM) proteins constitute a significant family of E3 ligases characterized by RING domains, B-box domains, and coiled-coil regions [10, 11]. A growing body of research has identified several E3 ligases as critical regulators of tumor immune responses [12]. Certain drugs targeting E3 ubiquitin ligases have demonstrated promising effects in preclinical and clinical antitumor treatments [13]. However, the specific roles of E3 ligases in cancer cells, particularly in the context of drug resistance in cancer immunotherapy, remain inadequately understood.

Immune evasion has been closely linked to the activation of the Nuclear Factor NF-Kappa-B (NF-κB) pathway across various tumor types [14]. The NF-κB pathway also significantly influences the tumor immune landscape by stimulating the production of cytokines and other pro-inflammatory mediators from cancer cells. These molecules modulate the immune system via paracrine interactions [15]. Proinflammatory cytokines produced in the tumor microenvironment facilitate the suppression of antitumor immunity and enhance tumor cell survival. In recent years, significant emphasis has been placed on the classical and alternative NF-κB pathways as promising molecular targets for enhancing the antitumor activity of checkpoint inhibitors [15, 16]. Consequently, a comprehensive exploration of NF-κB pathways in the context of tumor immune checkpoints may reveal alternative strategies for bolstering antitumor immunity.

Tumors exert control over the immune microenvironment by recruiting diverse cell types that aid in niche formation, metastatic progression, and resistance to therapy [17]. The abnormal differentiation and function of myeloid cells represent a hallmark of cancer. Tumors commonly exhibit an accumulation of relatively immature and pathologically activated MDSCs known for their potent immunosuppressive activity [18]. The recruitment of MDSCs to the tumor bed is mediated by the expression of CXCR2 ligands, including CXCL1 and CXCL2 [18]. Given the crucial role of MDSCs as immune checkpoint inhibitors (ICIs) and their association with resistance to PD-1 or PD-L1 inhibitors, the combination of MDSC inhibitors with PD-1/PD-L1 inhibitors holds the potential to significantly enhance antitumor effects compared to the use of PD-1/PD-L1 inhibitors alone [19]. Therefore, the co-administration of MDSC inhibitors alongside PD-1/PD-L1 inhibitors may achieve superior antitumor outcomes compared to using PD-1/PD-L1 inhibitors in isolation. However, our understanding of the regulators governing MDSC chemotaxis and function remains limited. Thus, the quest to elucidate the molecular signals driving MDSC development and homeostasis remains of paramount importance.

Herein, we demonstrated that TRIM28 is pivotal in promoting the chemokine-driven recruitment of MDSCs through the NF-kB signaling pathway within the tumor microenvironment. This recruitment suppresses infiltrating activated CD8^+^T cells, resulting in disease progression and resistance to anti-PD-1 therapy. Mechanistically, TRIM28 interacts with RIPK1 and facilitates the K63-linked polyubiquitination of RIPK1, ultimately activating the NF-κB signaling pathway. Furthermore, the activation of NF-κB stimulates the production of the potent chemokine CXCL1, which, in turn, facilitates the recruitment of MDSCs to the tumor site, ultimately leading to the suppression of CD8^+^T cell infiltration. Our findings underscore the potential of pharmacologic inhibition of TRIM28 as a promising strategy to counter tumor-induced immune tolerance and enhance the effectiveness of PD-1 blockade.

## Materials and methods

### Cell lines and cell culture

The lung cancer cell lines H1299 and CMT-167 were obtained from the Cell Bank of Type Culture Collection of the Chinese Academy of Sciences Shanghai Institute of Biochemistry and Cell Biology. Human embryonic kidney HEK293T cells were purchased from the American Type Culture Collection (ATCC) and maintained in our laboratory. All cells were cultured in Dulbecco’s modified Eagle’s medium (DMEM) supplemented with 10% fetal bovine serum (FBS) at 37 °C in a humidified atmosphere with 5% CO_2_. Cell lines were authenticated using short tandem repeat (STR) fingerprinting and tested negative for mycoplasma contamination prior to experimentation.

### Western blot and immunoprecipitation assays

Tumor tissue or cells were homogenized and lysed in RIPA buffer supplemented with a protease inhibitor cocktail and Phenylmethylsulfonyl Fluoride (Sigma). Protein samples were separated by SDS-PAGE and transferred onto PVDF membranes (Bio-Rad). Monoclonal/polyclonal primary antibodies and appropriate HRP-conjugated secondary antibodies were employed for Western blotting. Protein visualization was achieved using ECL-Plus. For immunoprecipitation (IP), cells were lysed, and protein concentrations were quantified. Primary antibodies were added and incubated overnight at 4 °C with rotation. Protein G beads were added and incubated for 3 h at 4 °C with rotation. The resin was centrifuged, washed three times with lysis buffer, and analyzed by immunoblotting.

The following antibodies were used: TRIM28 (Cat: PA5-27648, Invitrogen), RIPK1 (Cat: PA5-20811, Invitrogen), Phospho-IκBα (Cat: #2859, Cell Signaling Technology), IκBα (Cat: #4814, Cell Signaling Technology), Phospho-IKKα/β (Cat: #2697, Cell Signaling Technology), IKKα (Cat: #2682, Cell Signaling Technology), IKKβ (Cat: #8943, Cell Signaling Technology), anti-Flag (Cat: #14,793, Cell Signaling Technology), anti-Ubiquitin (Cat: #3936, Cell Signaling Technology), anti-K48-linkage Specific Polyubiquitin (Cat: #8081, Cell Signaling Technology), and anti-K63-linkage Specific Polyubiquitin (Cat: #5621, Cell Signaling Technology).

### Ubiquitination assay

For in vivo ubiquitination assay, 293T cells were transfected with the indicated plasmids. Plasmid transfections were performed using jetPRIME (Polyplus) according to the manufacturer’s protocol. At 24 h following transfection, cells treated with10µm MG132 (Sigma) for 6 h to inhibit the proteasome function and lysed in denaturing buffer A (6 M guanidine-HCl, 0.1 M Na2HPO4/NaH2PO4, and 10mM imidazole). The cells were lysed and immunoprecipitated for 4 h at 4 °C with Protein G Magnetic beads loaded or bound with anti-HA antibodies according to immunoprecipitation assay described above. The immunoprecipitated proteins were subjected to immunoblotting analysis with antibody against Ubiquitin.

### Reverse transcription quantitative PCR (RT-qPCR) analysis

Total RNA was extracted from tumor or normal tissues or cells using TRIzol reagent (Invitrogen), and converted to cDNA using the PrimeScript™ RT Master Mix (Takara) according to the manufacturer’s recommendations. Quantitative PCR (qPCR) was performed with One-Step TB Green PrimeScript RT-PCR Kit II (Takara), according to the instructions from the manufacturer. The value obtained for each gene was normalized to that of the gene encoding GAPDH. Mouse S100A9 (Cat: MP200308), human (Cat: HP100763) and mouse (Cat: MP200180) CXCL1, human (Cat: HP104854) and mouse (Cat: MP200388) CCL2, and human GAPDH (Cat: HP100003) and mouse GAPDH (Cat:MP200537) qPCR Primer Pair were purchased from Sino Biological Inc. The primers for mouse iNOS: 5′-GAGACAGGGAAGTCTGAAGCAC-3′ (forward) and 5′- CCAGCAGTAGTTGCTCCTCTTC-3′ (reverse) and Arginase-1: 5′- CATTGGCTTGCGAGACGTAGAC-3′ (forward) and 5′-GCTGAAGGTCTCTTCCATCACC-3′ (reverse).

### Plasmid constructs and RNA interference

Human HA-TRIM28, Flag-RIPK1(Cat: HG11981-NY), expression plasmid were purchased from Sino Biological Inc. Scrambled, human TRIM28 short hairpin RNAs (shRNAs), mouse TRIM28 lentiviral shRNAs viral particles, and mouse TRIM28 lentiviral particles was obtained from Shanghai Genechem Co., Ltd. (Shanghai, China). To elucidate the role of TRIM28 as an E3 ligase, we generated an E3 ligase-defective TRIM28 by using the KOD-Plus-Mutagenesis kit (Toyobo, cat: SMK-101) and verified by performing DNA sequencing. Plasmid transfection was performed using the Lipofectamine 3000 transfection reagent (Thermo Fisher, Cat. #L3000015) according to the manufacturer’s instructions. After 48 h, biological and biochemical experiments were performed.

### Immunohistochemistry (IHC) staining and scoring

Immunohistochemistry staining was performed according to the manufacturer’s instructions. Briefly, tissue slides were deparaffinized, rehydrated, and treated with 3% H_2_O_2_ solution for 15 min at room temperature to block endogenous peroxidase activity. Slides were then subjected to antigen retrieval in EDTA buffer (pH 9.0) at 96 °C for 20 min. After blocking with 10% goat serum, slides were incubated with primary antibodies at 4 °C overnight. The following antibodies were used: TRIM28 (Cat: PA5-27648, Invitrogen), CXCL1(Cat: PA5-86508, Invitrogen), anti-CD8(Cat: ab237723, abcam), and anti-S100A8 + S100A9(Cat: ab288715, abcam). Following incubation with secondary antibodies and development with 3,3-diaminobenzidine, the final IHC score was calculated based on staining extent and intensity. Staining extent was scored based on the percentage of positively stained cells (0 to 3), while staining intensity was scored as 0 (negative), 1 (weak), 2 (moderate), or 3 (strong). Specimens with final scores ≥ 2 were considered to have high expression, while those with final scores < 2 were considered to have low expression. For immune cell markers where signal intensity was not considered, the percent positive cell score was calculated based on the percentage of positively stained cells.

### Luciferase assays

Cells were cultured in 12 well plates, and then transiently transfected with NF-κB luciferase reporter plasmid (1 µg) and Renilla plasmid (100ng). At 48 h post-transfection, luciferase activities were measured using the Dual-Luciferase Reporter Assay System (Promega) according to the manufacturer’s protocols.

### Enzyme-linked immunosorbent assay (ELISA)

The CXCL1 concentrations in the cell supernatants were measured via ELISA according to the manufacturer’s instructions. The following ELISA kits were used in this study: mouse CXCL1 ELISA kit (R&D Systems) and human CXCL1/GRO alpha ELISA kit (R&D Systems).

### Immune Infiltration analysis

Tumor Immune Estimation Resource 2.0 (TIMER2.0, http://timer.cistrome.org/) web server is a comprehensive resource for systematical analysis of immune infiltrates across diverse cancer types [20]. It provides several different immune estimation algorithms including TIMER [20], CIBERSORT [21], quanTIseq [22], xCell [23], MCP-counter [24], EPIC [25]. For example, xCell (https://xcell.ucsf.edu/) calculates the enrichment fraction of each cell type signature in the bulk RNA data using the GSEA algorithm, then converts the enrichment fraction of each type to a cell type fraction, and finally adjusts the time relationship for the closely related cell types. This marker gene-based algorithm can only calculate a semi-quantitative score of cell-type enrichment, which has a linear correlation with the true cell proportion. At the same time, CIBERSORT (https://cibersortx.stanford.edu/) is used to characterise the expression data of 22 common immune infiltrating cells using microarray data to form LM22 files as a reference set. Based on the LM22 feature reference set, the expression matrix uploaded was deconvoluted to estimate immune cell abundance using linear support vector regression.

### In vivo drug response and Tumor growth assays

Lung cancer cell lines were transplanted into syngeneic wild-type C57BL/6 male mice or humanized mouse models by subcutaneous injection into the right flanks at 6 weeks of age. Tumors formed until volumes were ~ 150-200mm^3^ as measured by digital calipers before treatments were administered. Mice were treated weekly with 200 µg of anti-mouse PD-1 mAb (BE0146, Bio X cell) or anti-human PD-1 mAb (SIM0010, clone Pembrolizumab, Bio X Cell) by intraperitoneal injection. Control mice received solvent at a volume equal to the drug dosage at the indicated drug concentrations or 200 µg of isotype control (Bio X Cell). Tumor growth measurement were performed with calipers on the days as indicated in figures. Tumor sizes were calculated by using the formula: V = (S^2^× L)/2, where V is the volume, S is the shortest diameter, and L is the longest diameter.

### Humanized mouse models

huHSC-NOG-EXL mice were purchased from Charles River Laboratories. These mice received intravenous injections of 5 × 10^4^ human cord blood-derived CD34^+^ hematopoietic stem cells 12–24 h after sub-lethal whole-body gamma irradiation (150–170 cGy/animal) at 4–5 weeks of age. Twelve weeks post-engraftment, humanized mice reconstituted with over 45% human CD45 + cells were used for tumor studies. Human H1299 cells, with or without TRIM28 depletion, were injected subcutaneously to establish tumors.

### MDSC isolation and migration assay

Mouse MDSCs (CD11b^+^Gr1^+^) were isolated from the spleens of CMT-167 tumor-bearing C57BL/6 mice using the EasySep™ Mouse MDSC (CD11b^+^Gr1^+^) Isolation Kit (STEMCELL). Isolated MDSCs (1 × 10^5^ cells/well) were seeded in the top chamber of a transwell (pore size: 0.8 μm), and the bottom chamber was filled with conditioned medium (CM, without FBS) from CMT-167 cells with or without TRIM28. After a 4-hour incubation, MDSCs that migrated to the bottom chamber were counted.

### Immunofluorescence

Paraffin-embedded samples were sectioned at 4 μm thickness. Antigen retrieval was performed in an antigen unmasking solution in a pressure cooker (95 °C for 30 min in Antigen Unmasking Solution (Vector Laboratories, Cat# H-3300). Sections were blocked in PBS containing 2% bovine serum albumin for 1 h at room temperature. For dual immunofluorescence staining, slides were incubated with a mixture of two primary antibodies overnight at 4 °C. Primary antibodies used were rabbit anti-TRIM28(Invitrogen, Cat#PA5-27648), mouse anti-CD14(Invitrogen, Cat# 14-0149-82), and rabbit anti-p-p65(Invitrogen, Cat#MA5-15160). After washing with cold PBS, slides were incubated with a mixture of two secondary antibodies raised in different species for 1 h at room temperature in the dark. The following secondary antibodies were used: Alexa Fluor 594 labeled anti-rabbit (Invitrogen, Cat# A-11,012), and Alexa Fluor 488 labeled anti-mouse (Invitrogen, Cat# A32723). Slides were counterstained with DAPI and examined by fluorescence microscopy.

### T-cell suppression assay

CD11b^+^Gr1^+^ MDSCs were sorted from tumors of tumor-bearing mice using an EasySep™ Mouse MDSC (CD11b^+^Gr1^+^) Isolation Kit. CD8^+^ T cells were isolated from the spleen of naive mice using an EasySep mouse CD8^+^T cell isolation kit (STEMCELL) according to the manufacturer’s instructions and were labeled with 1µM carboxyfluorescein succinimidyl ester (CFSE) (BioLegend). T cells were stimulated with anti-CD3 (1 µg/mL; Bio-Legend) and anti-CD28 (1 µg/mL; BioLegend), incubated at 10^5^ cells per well, and co-cultured with sorted MDSCs at different ratios for 72 h. CD8^+^T cell proliferation by CFSE was measured using FACS analysis.

### Flow cytometry

Single-cell suspensions of tumor-burdened lungs were re-suspended in FACS buffer (PBS, 1%BSA), blocked by non-specific staining with Fc block (anti-mouse CD16/32 mAb; BD Biosciences), The following antibodies were acquired from Biolegend and used in the flow cytometry analysis: BV605 anti-mouse CD45 (clone 30-F11), PE anti-mouse CD3 (clone 17A2), PE/Cy7 anti-mouse CD4 (clone RM4-5), BV711 anti-mouse CD8α (clone 53 − 6.7), BV421 anti-mouse Ly-6 C (clone HK1.4), PE anti-mouse Gr-1 (clone RB6-8C5), PE/Cy5 anti-mouse CD11b (clone M1/70), PE/Cy7 anti-mouse Ly-6G (clone 1A8), BV711 anti-mouse IFN-γ (clone XMG1.2), BV421 anti-mouse CD8α (clone 53 − 6.7), PE/Cy5 anti-mouse CD3 (clone 17A2). Flow cytometry data were acquired on a FACS Celesta flow cytometer and data was analysed with FlowJo V10.

### Induction of tumorigenesis in KP mouse model

All murine procedures were approved by the Institutional Animal Care and Use Committee at Wannan Medical College. *Kras*^*LSL−G12D/+*^; *Tp53*^*fl/fl*^ (KP) mice were purchased from the Jackson Laboratory. At 6–8 weeks of age, anesthetized mice were infected with 10^7^ infectious units/ml of Lenti-TRIM28-Cre or Lenti-GFP-Cre lentiviruses (Obio Technology, Shanghai) by intranasal or intratracheal instillation. The survival of mice was recorded until the end of the study. Alternatively, the lungs were removed for subsequent histological analysis at the indicated time points after infection.

### Statistical analysis

Statistical analyses were performed using Prism 9.0 (GraphPad). Comparisons between categorical variables were executed using the Chi-square test. Correlations between two continuous variables, when applicable, were assessed with the Spearman correlation test. Survival curves were constructed using the Kaplan-Meier method and analyzed through log-rank tests. For parametric data, two-tailed unpaired/paired Student’s t-tests were employed to perform statistical tests between the two groups. In cases involving three or more groups, one-way ANOVA tests were carried out, followed by Tukey’s post-hoc test for multiple comparisons. Sample sizes for in vivo mouse studies were determined based on the number of mice required to establish statistical significance, considering variability within study arms and previous experience documented in publications [26]. A p-value less than 0.05 was statistically significant.

## Results

### TRIM28 expression positively correlated with suppressive MDSC cell infiltration in NSCLC

**Fig. 1:**
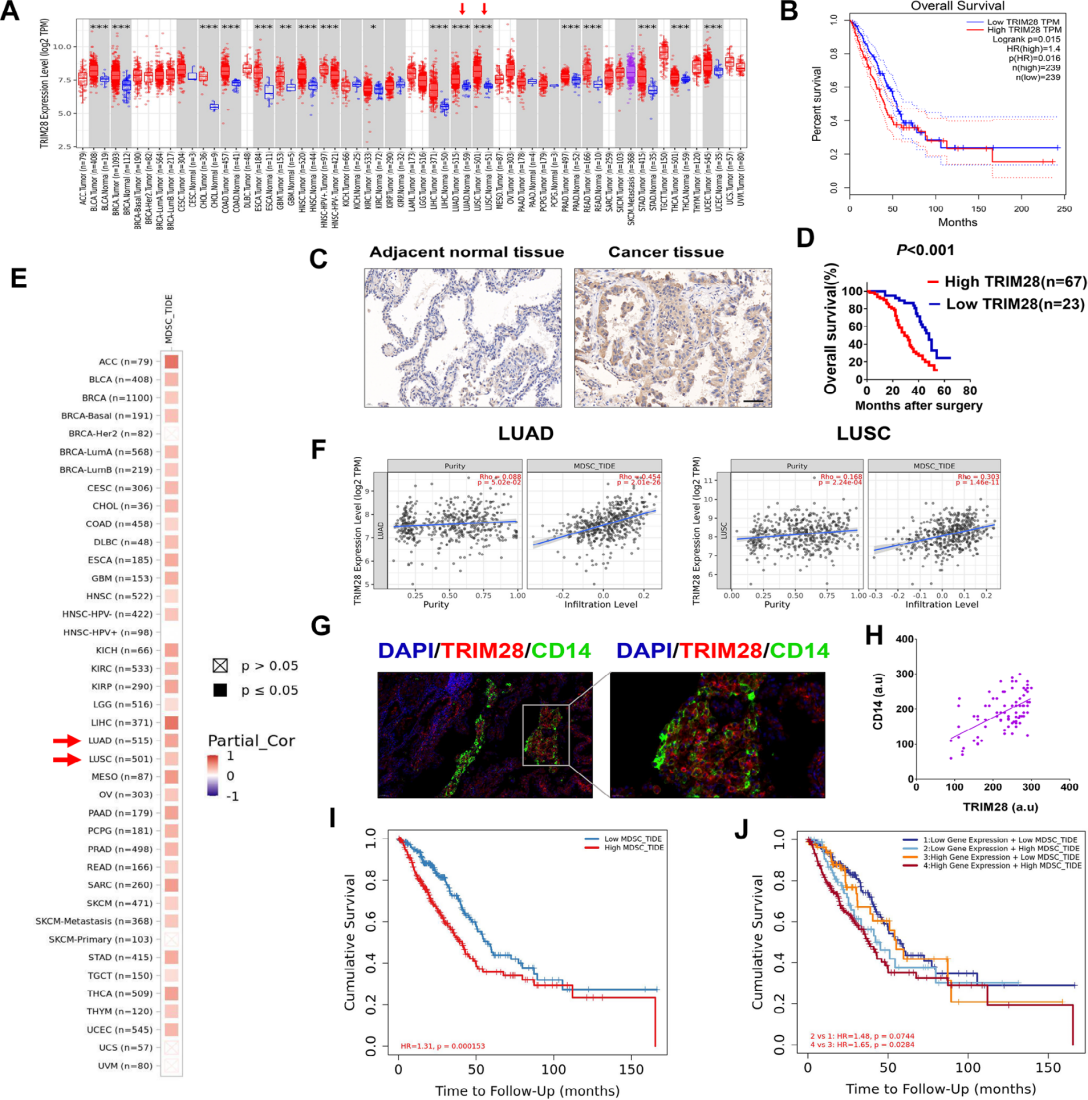
TRIM28 plays a mechanistic role in tumor progression by recruiting MDSCs into the tumor microenvironment. (**A**) TRIM28 expression levels in different tumor types from TCGA database were analyzed by TIMER2.0 (*p < 0.05, **p < 0.01, ***p < 0.001). (**B**) Survival analysis comparing the high and low expression of TRIM28 in lung adenocarcinoma according to TCGA dataset by using the website GEPIA 2 (http://gepia2.cancer-pku.cn/#survival). The high or low expression of TRIM28 were divided according to 50% of the total sample. (**C**) Immunohistochemical analysis of TRIM28 protein levels in NSCLC samples on tissue microarrays. Representative examples of TRIM28 expression in adjacent non-cancerous lung tissues, NSCLC tissues are shown. The scale bars represent 100 μm. (**D**) Overall survival analysis of patients with NSCLC stratified by the TRIM28 expression level in 90 samples. Kaplan-Meier survival analysis indicating a significant association between higher TRIM28 expression and poorer OS in NSCLC. (**E**) The correlations of TRIM28 expression and MDSCs infiltration in pan-cancers were analyzed by TIMER2.0. (**F**) Correlation of TRIM28 expression, tumor purity, and MDSCs infiltration in TCGA lung adenocarcinoma (LUAD) and lung squamous cell cancer (LUSC). The expression of TRIM28 positively correlates with MDSCs infiltration in NSCLC. (**G**-**H**) Representative immunofluorescence staining of CD14 and TRIM28 in tissue from human lung adenocarcinoma and the correlation between TRIM28 and CD14 intensity. The expressions of TRIM28 and CD14 were measured with mean fluorescence intensities (MFIs) (in arbitrary units, a.u.), respectively. The pearson correlation between TRIM28 and CD14 expression (n = 90; *p* < 0.01, r = 0.567). Scale bars: 50 μm. (**I**-**J**) Cox regression analyses using data from TCGA indicated that high MDSC infiltration was significantly associated with poorer prognosis in NSCLC. Furthermore, elevated TRIM28 expression and a high proportion of MDSCs significantly correlated to poorer OS compared to their counterparts, strongly suggesting that TRIM28 influenced patient prognosis through an immune-related mechanism. Split infiltration percentage of patients: 50% (**I**). Split expression percentage of patients: 50% and split infiltration percentage of patients: 50% (**J**)

Using data from the Cancer Genome Atlas (TCGA) (https://www.cancer.gov/ccg/research/genome-sequencing/tcga) databases, we examined TRIM28 expression across various cancer types and their corresponding non-tumor tissues. Our analysis revealed a significant elevation of TRIM28 expression in cancer samples compared to adjacent non-tumor tissues across multiple cancer types. Notably, TRIM28 mRNA levels were substantially higher in NSCLC tissues than in non-tumor tissues (Fig. 1A, Supplementary Table 1). Kaplan-Meier analysis indicated a significant negative correlation between high TRIM28 expression and the overall survival (OS) of NSCLC patients (Fig. 1B). To further investigate TRIM28’s role in NSCLC development, we conducted immunohistochemistry analysis on an NSCLC tissue microarray (Fig. 1C). Analysis of TRIM28 expression levels in conjunction with clinicopathological features revealed that high TRIM28 expression correlated with larger tumor size, lymph node metastasis, distant metastasis, and advanced pathological stage (Supplementary Table 2). Moreover, NSCLC patients with high TRIM28 expression levels exhibited a poorer prognosis (Fig. 1D). These findings collectively suggest that TRIM28 is upregulated in NSCLC tumor tissues and is inversely associated with the OS of NSCLC patients.

Aberrations in certain signaling pathways within tumor cells not only drive tumor cell proliferation and survival but also dictate the development of pro-tumorigenic microenvironments [27]. There is now substantial evidence indicating that oncogene expression is correlated with immune cell composition in human tumors [27]. Our findings prompted us to explore the role of TRIM28 in tumor progression and its impact on the tumor microenvironment. Consequently, we aimed to investigate the relationship between TRIM28 and immune cells through correlation analysis using the TIMER2.0 database. Interestingly, our results from TIMER2.0 analysis revealed a significant association between TRIM28 expression and MDSCs infiltration across various cancer types (Fig. 1E). This correlation was consistently observed in NSCLC (Fig. 1F). MDSCs in cancer patients were characterized by the expression of CD14, CD11b, CD33, and Arg1, along with low or absent expression of HLA-DR. When analyzing the tumor microenvironment of TRIM28-expressing NSCLC tumors, we observed an increase in CD14^+^ cells. Immunofluorescence staining demonstrated a positive correlation between the intensity of TRIM28 and CD14 expression in these tumors (Fig. 1G-H). Additionally, multivariate Cox analysis revealed an increased proportion of MDSCs was associated with a poor prognosis in NSCLC patients (Fig. 1I).

In addition, we analyzed the association between MDSC levels and OS in NSCLC patients, considering high or low TRIM28 expression using TIMER2.0. In the group of cancer patients with low TRIM28 expression, there was no significant difference in OS between those with low and high MDSC infiltration. However, in the group of cancer patients with high TRIM28 expression, higher levels of MDSCs were associated with worse patient survival, suggesting that elevated TRIM28 levels might enhance the immune-suppressive effect of MDSCs (Fig. 1J). These findings suggest an association between TRIM28 expression and immunosuppressive MDSCs in cancer. In summary, TRIM28 exhibits high expression in NSCLC tumor tissues and positively correlates with MDSC infiltration in the tumor microenvironment, potentially contributing to enhance tumor progression.

### TRIM28 suppression sensitizes lung tumors to PD-1 blockade

Based on our observations that TRIM28 expression is strongly associated with MDSC infiltration in NSCLC, we hypothesized that TRIM28 promotes the chemotactic recruitment of MDSCs into the tumor microenvironment. To assess the effect of TRIM28 on MDSC infiltration in NSCLC, we conducted immunohistochemistry on MDSCs from syngeneic murine tumors. CMT-167 cells, with or without TRIM28 knockdown, were subcutaneously implanted into C57BL/6J mice. As expected, depletion of TRIM28 led to a profound reduction in MDSCs, whereas overexpression of TRIM28 increased MDSC infiltration in the tumor microenvironment (Supplementary Fig. 1). These results support our initial findings in patient tumor samples and emphasize the critical relationship between TRIM28 expression and MDSCs in cancer.

The effectiveness of checkpoint immunotherapy in NSCLC largely depends on the tumor microenvironment. Previous research has demonstrated that PD-1 blockade can transiently reduce tumor growth and metastasis in syngeneic tumor models by increasing CD8^+^ tumor-infiltrating lymphocytes (TILs) and decreasing exhausted CD8^+^TILs. However, increased MDSC infiltration is associated with treatment resistance to PD-1 blockade, reduced CD8^+^TILs, and increased exhausted CD8^+^TILs. Therefore, we investigated whether targeting TRIM28 in lung cancer could enhance the efficacy of anti-PD-1 therapy.

**Fig. 2:**
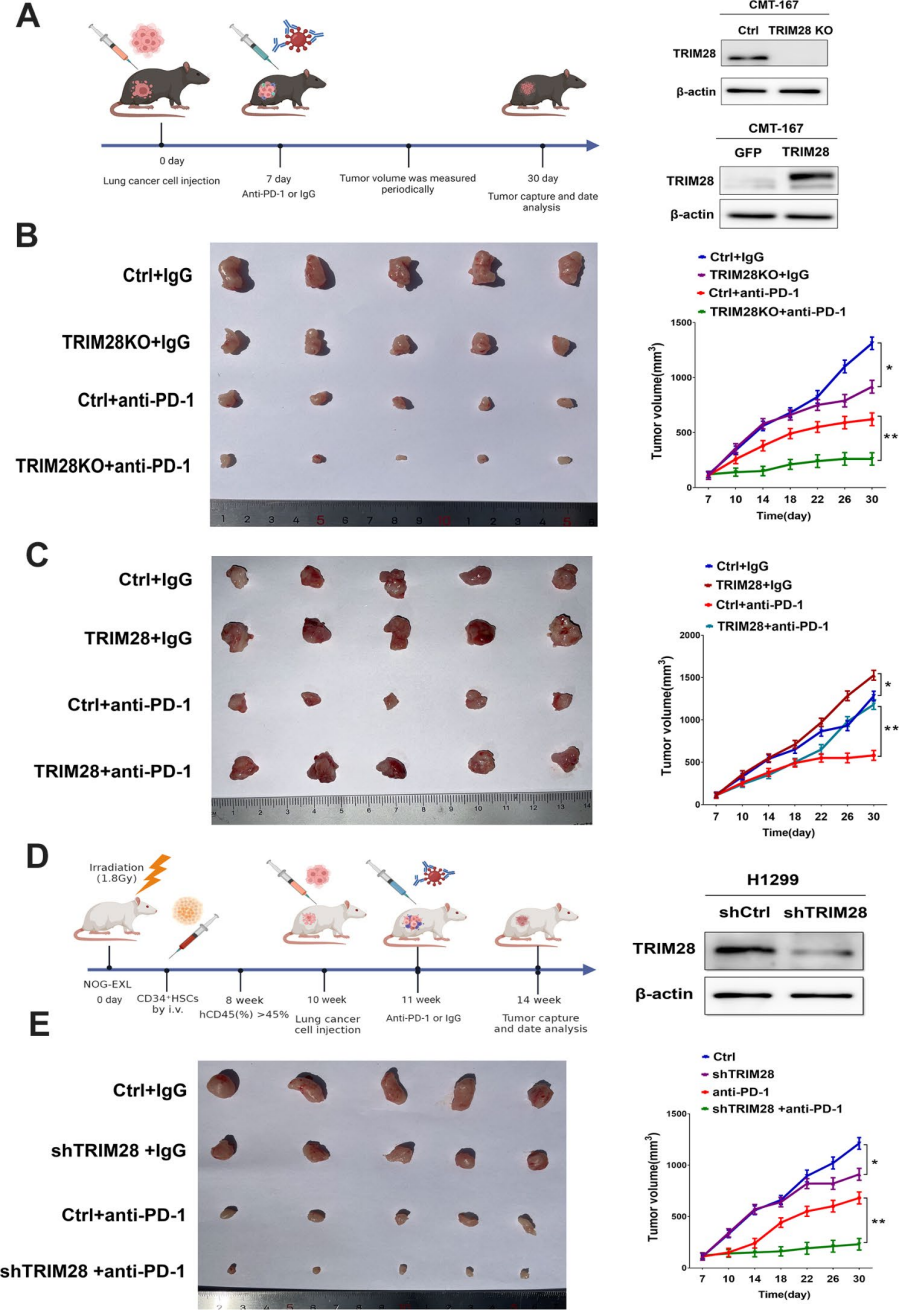
TRIM28 inhibition enhances anti-PD-1 therapy in a syngeneic lung cancer model. (**A**) The schematic of tumor inoculation and treatment in mice. Western blot validated TRIM28 knockout or overexpression in CMT-167 cells. (**B**) C57BL/6J mice were subcutaneously injected with shControl or shTRIM28 CMT-167 cells and weekly with anti-PD-1 (200 µg/mouse) or isotype control (200 µg/mouse) by intraperitoneal injections starting on day 7 post tumor cell injection. Tumor growth curves and tumor weight were shown. n = 5 mice per treatment group. *p < 0.05; **p < 0.01. (**C**) C57BL/6J mice were subcutaneously injected with Control or TRIM28 CMT-167 cells and weekly with anti-PD-1 (200 µg/mouse) or isotype control (200 µg/mouse) starting on day 7 post-tumor cell injection. Tumor growth curves and tumor weight were shown. n = 5 mice per treatment group. *p < 0.05; **p < 0.01. (**D**) Experimental strategy. (**E**) Humanized huHSC-NOG-EXL mice were injected with shControl or shTRIM28 H1299 cells and treated with anti-PD-1(200 µg/mouse). Control animals were treated with isotype control. Tumor growth curves and tumor weight were shown. The treatment schema is as in (**D**). n = 5 mice per treatment group. Statistics were calculated using a one-way ANOVA post hoc Tukey test. *p < 0.05; **p < 0.01

In syngeneic lung cancer models, we evaluated the impact of TRIM28 inhibition on the efficacy of anti-PD-1 therapy. As expected, the knockdown of TRIM28 alone decreased tumor growth. Both TRIM28 knockout and anti-PD-1 treatment alone reduced tumor size and weight compared to the control. Importantly, the combination of TRIM28 knockdown and anti-PD-1 significantly reduced tumor volume and weight, leading to a sustained reduction in tumor size throughout the treatment (Fig. 2A-B). In contrast, TRIM28 overexpression conferred resistance to anti-PD-1 therapy (Fig. 2C), further highlighting the significance of TRIM28 expression in maintaining sensitivity to anti-PD-1 therapy in NSCLC. To further investigate whether the therapeutic benefits of combined TRIM28 inhibition and anti-PD-1 extend to human NSCLC, we utilized a human lung adenocarcinoma cell line, H1299, in a humanized huHSC-NOG-EXL mouse model. Consistently, the combination of TRIM28 inhibition with anti-PD-1 resulted in sustained control of tumor progression (Fig. 2D-E). These findings suggest that TRIM28 promotes resistance to anti-PD-1 therapy in cancer by recruiting MDSCs into the tumor microenvironment.

### TRIM28 is required for K63-linked ubiquitination of RIPK1

**Fig. 3:**
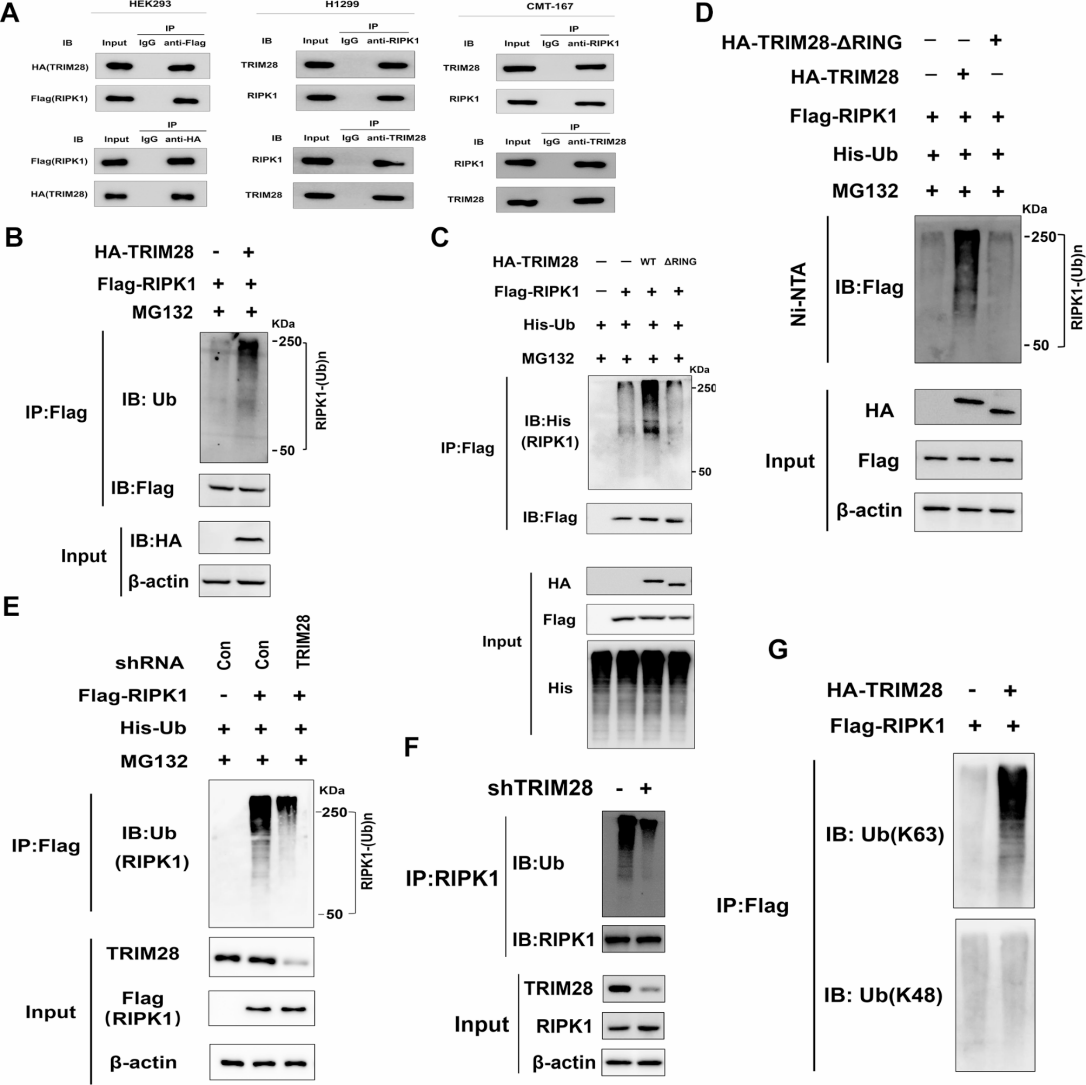
TRIM28 is required for K63-linked ubiquitination of RIPK1. (**A**) Ectopic HA-TRIM28 interacts with Flag-RIPK1 in 293T cells. Endogenous TRIM28 interacts with endogenous RIPK1 in CMT-167 and H1299 cells. (**B**) HEK293 cells were transfected with HA-TRIM28 and Flag-RIPK1 as indicated. Cell lysates were immunoprecipitated with anti-Flag and immunoblotted with anti-ubiquitin, anti-RIPK1, or anti-HA as indicated. (**C** and **D**) RIPK1 ubiquitination is increased upon overexpression of TRIM28-WT but not the ΔR mutant. 293T cells were transfected with HA-TRIM28 WT or ΔR mutant, and the cell lysates were subjected to immunoprecipitation using anti-Flag antibodies (**C**) or Ni-NTA pull-down under denaturing conditions (**D**), followed by immunoblotting with the indicated antibodies (**E**) RIPK1 ubiquitination was decreased upon TRIM28 depletion. 293T cells were co-transfected with the indicated plasmids or shRNAs, and Flag-RIPK1 was immunoprecipitated and analyzed by immunoblotting. (**F**) H1299 cells were transfected with control or TRIM28 shRNA, and endogenous RIPK1 was immunoprecipitated and analyzed for ubiquitination (**G**) TRIM28 modified RIPK1 by K63-linked ubiquitination. Cell lysates prepared in (**C**) were immunoprecipitated with anti-Flag and blotted with anti-ubiquitin K63 and anti-ubiquitin K48

Structure-function evaluations have demonstrated that TRIM28’s tumor-promoting functions rely on its E3 ubiquitin ligase activity. To further investigate which protein TRIM28 interacts with and how this interaction affects the target protein’s physiological function, we conducted affinity capture-western assays, which confirmed that TRIM28 can interact with RIPK1 [28]. Subsequently, we conducted co-immunoprecipitation (Co-IP) analysis by co-transfecting Flag-RIPK1 and HA-TRIM28 into HEK293T cells. After immunoprecipitation with anti-Flag magnetic beads, we detected HA-TRIM28 and vice versa (Fig. 3A). Additionally, endogenous TRIM28 was found to bind endogenous RIPK1 in both CMT-167 and H1299 cells (Fig. 3A).

Since TRIM28 possesses a RING finger domain, it has been considered an E3 ubiquitin ligase. Therefore, we investigated whether TRIM28 functions as an E3 ubiquitin ligase for RIPK1. We overexpressed Flag-RIPK1 with or without HA-TRIM28 and examined RIPK1 ubiquitination using immunoblot analysis with a ubiquitin antibody. As depicted in Fig. 3B, cells expressing TRIM28 exhibited induction of Flag-RIPK1 ubiquitination.

Next, HEK293 cells were transfected with plasmids encoding HA-TRIM28 or a mutant form, HA-TRIM28-∆RING, alongside Flag-RIPK1 and His-ubiquitin. Cells with HA-TRIM28 expression displayed increased ubiquitination of Flag-RIPK1 compared to control vector-transfected cells. Notably, RIPK1 ubiquitination was not observed in cells expressing the TRIM28-ΔRING mutant, indicating that TRIM28 potentiated RIPK1 ubiquitination through its E3 ligase activity (Fig. 3C-D). Consistently, the depletion of TRIM28 significantly inhibited RIPK1 ubiquitination (Fig. 3E). Moreover, TRIM28-depleted H1299 cells exhibited decreased ubiquitination of endogenous RIPK1 (Fig. 3F). Taken together, these findings indicate that TRIM28 is essential for RIPK1 ubiquitination, with RIPK1 being a substrate for TRIM28.

To determine the type of ubiquitin chain on RIPK1 modified by TRIM28, we performed an in vivo ubiquitination assay. Immunoblot analysis with a ubiquitin lysine (K) 63 antibody demonstrated that TRIM28 overexpression increased K63-linked ubiquitination of RIPK1. Conversely, TRIM28 overexpression did not affect the K48-linked ubiquitination of RIPK1, as determined by immunoblot analysis with a ubiquitin K48 antibody (Fig. 3G). These results suggest that TRIM28 primarily catalyzes the K63-linked ubiquitination of RIPK1. In summary, TRIM28 is an E3 ligase for K63-linked ubiquitination of RIPK1.

### TRIM28 activates the NF-κB signaling pathway

**Fig. 4:**
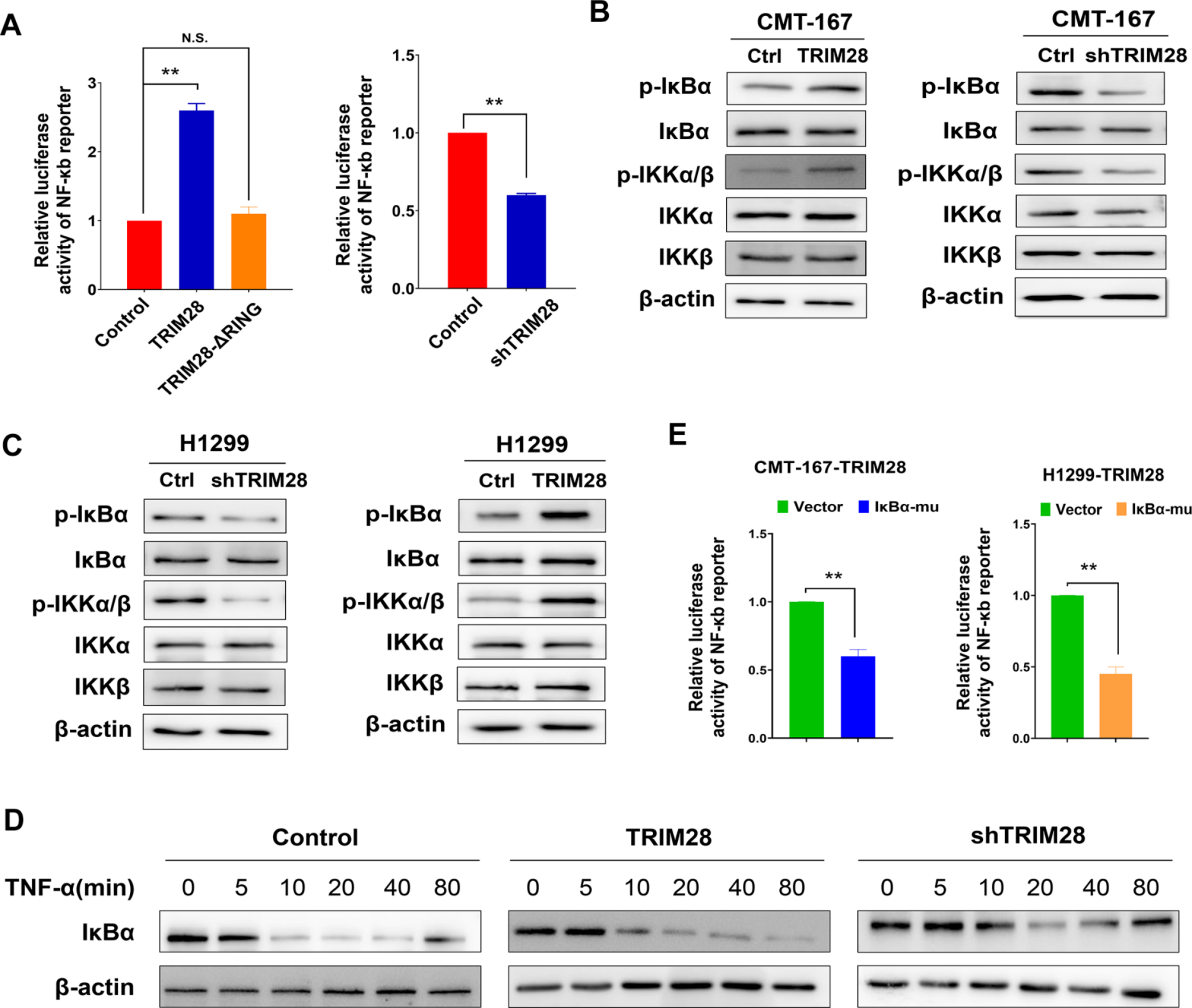
TRIM28 activates NF-κB signaling. (**A**) HEK293 cells were co-transfected with the indicated plasmids along with pNF-κB-luc plasmids or the control-luciferase plasmid and subjected to a reporter assay. The luciferase assay showed that TRIM28, but not the ΔR mutant, induced the activation of NF-κB signaling. n.s., not significant; **p < 0.01. (**B**) p-IκBα, IκBα, p-IKKα/β, IKKα, IKKβ, p-p65, p65, TIRM28, and β-actin detected by western blot in TRIM28-knockdown and TRIM28-overexpressed CMT-167 cells. (**C**) p-IκBα, IκBα, p-IKKα/β, IKKα, IKKβ, p-p65, p65, TIRM28, and β-actin detected by western blot in TRIM28-knockdown and TRIM28-overexpressed H1299 cells. (**D**) Western blotting analysis of IκBα expression in the indicated cells treated with TNF-α (10ng/ml). β-actin is used as a loading control. (**E**) Assay of NF-κB luciferase reporter gene activity in TRIM28-overexpressing CMT-167 and H1299 cells transfected with vector or the IκBα dominant negative mutant (IκBα-mu). **p < 0.01. In (**A**) to (**C**), and (**E**), analyses were done in triplicate. Data represent mean ± SEM from each of three independent experiments. Statistics calculated using one-way ANOVA post hoc Tukey test for multi-group or two-tailed Student’s t-test for two-group comparisons

It has been reported that TRIM28 plays a crucial role in activating the NF-κB signaling pathway. When TNF-α activates the NF-κB pathway, TRAF2 and RIPK1 are rapidly recruited to the membrane TNF-α receptor to form complex I, leading to K63-linked ubiquitination, which is a crucial step in NF-κB pathway activation. Previous studies have highlighted the importance of K63-linked polyubiquitination of RIPK1 in NF-κB signaling activation, a conserved regulator of immune and inflammatory responses [15]. Our results demonstrated that TRIM28’s E3 ligase activity is essential for mediating the K63-linked ubiquitination of RIPK1, suggesting that TRIM28 may play a significant role in driving NF-κB signaling pathway activation. Consistent with these findings, luciferase assays indicated that the activity of the NF-κB luciferase reporter gene increased in cells overexpressing wild-type TRIM28 but not the TRIM28-ΔRING mutant. Conversely, silencing TRIM28 reduced reporter activity, emphasizing the necessity of TRIM28’s E3 ligase activity for NF-κB activation (Fig. 4A).

To further substantiate these results, we observed that TRIM28 overexpression substantially increased the phosphorylation of IκBα and IKKα/β while silencing TRIM28 significantly inhibited NF-κB signaling pathway activation in CMT-167 and H1299 cells (Fig. 4B-C, Supplementary Fig. 2). Additionally, we investigated whether TRIM28 facilitated sustained NF-κB activation by treating cells with TNF-α and assessing the protein levels of IκB. TRIM28-overexpressing cells exhibited longer-lasting reductions in IκB levels, while TRIM28-silenced cells exhibited shorter-lasting reductions (Fig. 4D). To further validate these findings, we blocked the NF-κB pathway by expressing an IκBα dominant-negative mutant (IκBα-mu) in TRIM28-overexpressing cells. As expected, the NF-κB pathway activation induced by TRIM28 overexpression was inhibited by IκBα-mu (Fig. 4E). Overall, these results illustrate that TRIM28 plays a pivotal role in NF-κB activation.

### TRIM28 induces CXCL1 expression via the NF-κΒ pathway

**Fig. 5:**
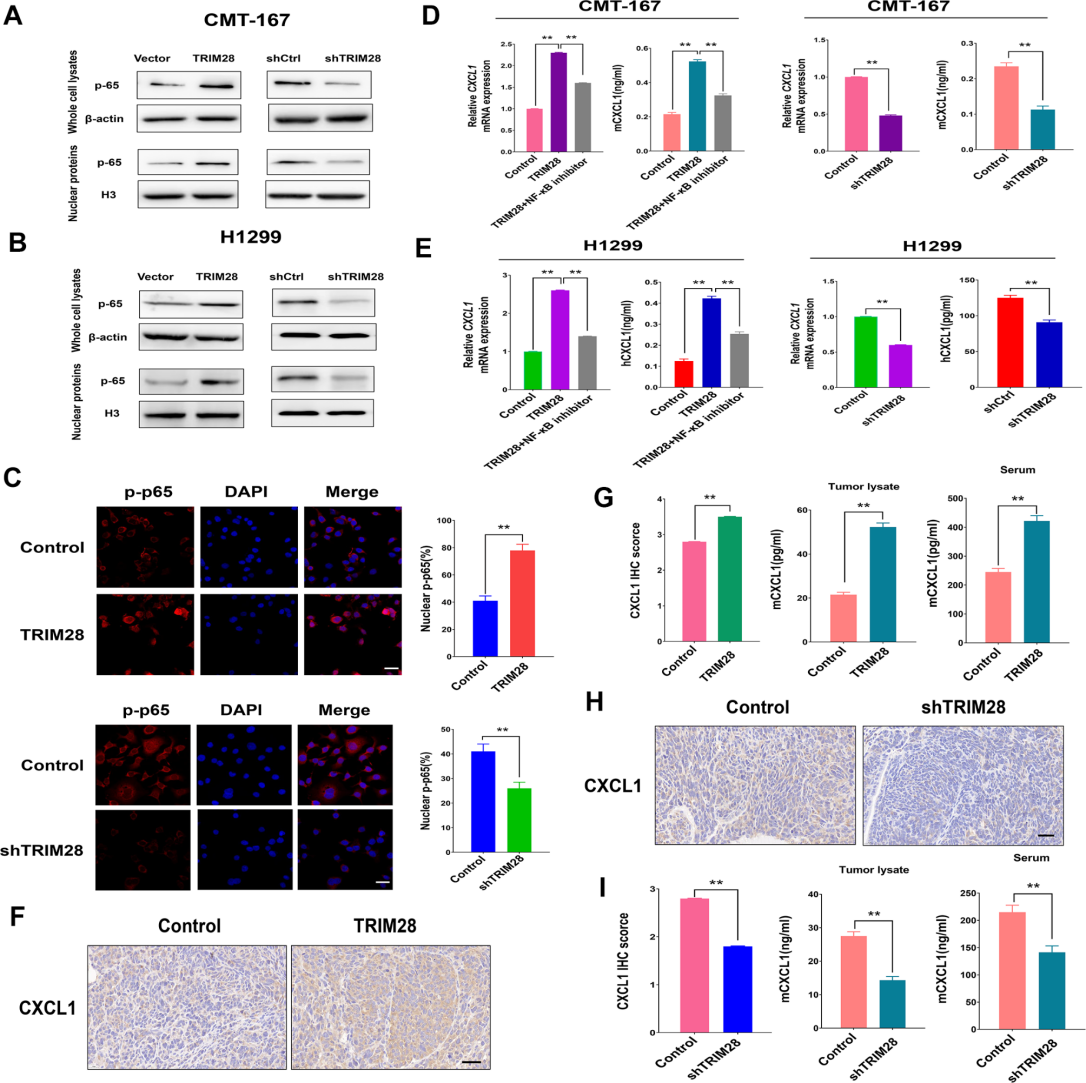
TRIM28 induces CXCL1 via the NF-κB pathway. (**A**-**B**) Expression of p65 was determined by western blot in CMT-167 or H1299 cells overexpressing wild-type TRIM28 or TRIM28 knockdown with shRNA. The whole cell lysates and nuclear protein extracts were subjected to immunoblotting analysis. Data are representative of results obtained in three independent experiments. (**C**) The immunofluorescence assays of p-65 were performed in CMT-167 cells treated with the indicated plasmids. DAPI (blue) was used as a nuclear counterstain. The quantification of nuclear p-65 positive staining in at least 200 counted cells was presented as percentage ± SEM. Statistics calculated using one-way ANOVA post hoc Tukey for multiple comparisons. (**D**-**E**) RT-qPCR determination of CXCL1 mRNA expression in TRIM28-knockdown and TRIM28-overexpressed CMT-167 or H1299 cells, treated with or without NF-κB inhibitor (BAY11-7085) at 10µM for 24 h. ELISA validation of levels of CXCL1 in cell culture supernatants from CMT-167 or H1299 cell culture. Data are representative of results obtained in three independent experiments. Statistics calculated using one-way ANOVA post hoc Tukey test for multi-group or two-tailed Student’s t-test for two-group comparisons. (**F**-**G**) Representative IHC staining and quantification for CXCL1 in the indicated tumor models are shown. Additionally, ELISA was performed to validate the levels of CXCL1 in tumor lysates and sera from CMT-167 syngeneic tumor models, including subcutaneous tumors and blood samples collected from mice bearing CMT-167 control and CMT-167-shTRIM28 tumors. The scale bar represents 50 μm. (**H**-**I**) Representative IHC staining and quantification for CXCL1 in the indicated tumor models are displayed. Additionally, ELISA was conducted to validate the levels of CXCL1 in tumor lysates and sera from CMT-167 syngeneic tumor models, including subcutaneous tumors and blood samples collected from mice bearing CMT-167 control and CMT-167-TRIM28 tumors. The scale bar represents 50 μm. Statistical analysis was performed using a two-tailed student’s t-test. **p < 0.01

Prior studies have shown that the NF-κB pathway activates CXCL1 transcription [29]. CXCL1 is a chemotactic factor that recruits MDSCs into the tumor microenvironment by binding to its receptor CXCR2 [30]. Therefore, we hypothesized that TRIM28-mediated NF-κB pathway activation might be responsible for enhancing CXCL1 expression. To explore this possibility, we analyzed nuclear phospho-p65 levels in lung cancer cell lines using western blot analysis. Phospho-p65, a key factor in the canonical NF-κB pathway, was decreased in TRIM28-depleted cell lines and increased in TRIM28-overexpressing cell lines (Fig. 5A-B, Supplementary Fig. 3). Subsequently, we examined phospho-p65 cellular localization in CMT-167 cells after modulating TRIM28 levels. Consistent with the western blot data, immunofluorescence results revealed a substantial increase in nuclear phospho-p65-positive staining in TRIM28-overexpressing cells compared to control cells, while TRIM28 knockdown attenuated nuclear phospho-p65 protein levels (Fig. 5C).

Next, we assessed whether TRIM28 alters CXCL1 expression in murine and human lung cancer cell lines. RT-qPCR confirmed that TRIM28 knockout suppressed CXCL1 mRNA levels in CMT-167 and H1299 cells. Conversely, ectopic expression of TRIM28 in lung cancer cells had the opposite effect (Fig. 5D-E). To further validate that TRIM28-mediated NF-κB activation promotes CXCL1 and CCL2 expression, we treated TRIM28-overexpressing cells with the NF-κB inhibitor, BAY11-7085. CXCL1 expression significantly decreased after NF-κB inhibitor treatment compared to controls (Fig. 5D-E). ELISA assays consistently revealed that TRIM28 knockout significantly reduced CXCL1 secretion in cell culture supernatants of CMT-167 and H1299 cells (Fig. 5D-E). Furthermore, elevated TRIM28 levels caused a corresponding increase in CXCL1 expression. Activating the NF-κB signaling pathway could also promote CCL2 expression, contributing to MDSC accumulation in the tumor microenvironment [31]. Similarly, upregulated TRIM28 expression promoted CCL2 expression, while downregulated TRIM28 expression significantly reduced CCL2 expression (Supplementary Fig. 4).

To corroborate these findings in vivo, we examined CXCL1 levels in tumor tissue, tumor lysates, and serum from a syngeneic CMT-167 tumor model. TRIM28 overexpression in CMT-167 tumors led to an upregulation of CXCL1 levels in tumor tissue, tumor lysates, and serum (Fig. 5F-G). Conversely, inhibiting TRIM28 significantly decreased CXCL1 expression (Fig. 5H-I). In summary, these results suggest that TRIM28 in tumor cells induces the activation of the NF-κB pathway, which contributes to the expression of CXCL1.

### TRIM28 recruitment and activation of MDSCs

**Fig. 6:**
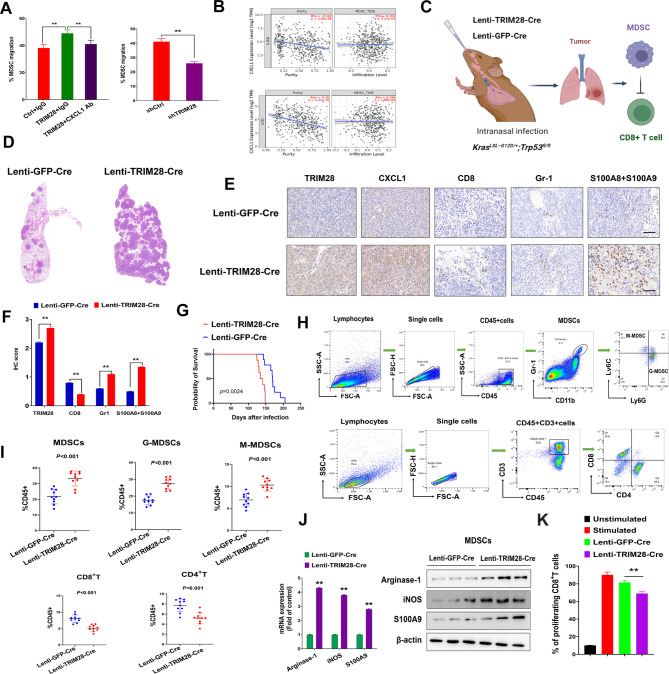
TRIM28 Promotes MDSCs recruitment in autochthonous tumors from KP mice. (**A**) Migration of MDSCs toward conditioned medium (CM) from CMT-167 cells were transfected with the indicated plasmids, treated with or without immunoglobulin G (IgG) control or CXCL1-neutralizing antibody. Data are representative of results obtained in three independent experiments. (**B**) Correlation of CXCL1 expression, tumor purity, and MDSCs infiltration in TCGA lung adenocarcinoma and lung squamous cell cancer. The expression of CXCL1 positively correlates with MDSCs infiltration in NSCLC. (**C**) Schematic representation of lentiviral TRIM28 or GFP overexpression in KP mice. KP mice were anesthetized with sodium pentobarbital followed by intranasal injection of Ad-Cre (1.5 × 10^6^ pfu/mouse). (**D**) Representative images of HE staining in tumor-burdened lungs of KP mice were analyzed. (**E**-**F**) Representative IHC (**E**) and IHC quantification (**F**) for immune cell markers (CD8, CD8^+^T cells; Gr-1, S100A8, S100A9, MDSC cells) in indicated in indicated tumor models. The scale bar represents 50 μm. Statistics calculated using a one-way ANOVA post hoc Tukey test. (**G**) Kaplan-Meier survival analysis of KP mice infected with Lenti-GFP-Cre or Lenti-TRIM28-Cre. Log-rank test. (**H**-**I**) Flow cytometry gating strategy of immune cells. Quantification of flow-cytometry data for CD8^+^T, CD4^+^T, MDSCs as a percentage of leukocytes (CD45^+^) in tumor-burdened lungs from indicated tumor models. (**J**) Real-time qPCR and western blotting showed expression of representative MDSCs immunosuppressive gene in the indicated cell lines. Data are representative of results obtained in three independent experiments. (**K**) Quantification of the proliferation of CFSE-labelled CD8^+^T cells cocultured with MDSCs from tumor-burdened lungs of KP or KP-TRIM28 mice, analyzed by flow cytometry. Data are representative of results obtained in three independent experiments. Statistics calculated using one-way ANOVA post hoc Tukey test for multi-group or two-tailed Student’s t-test for two-group comparisons. **p < 0.01

Next, we examined the role of the TRIM28-CXCL1 axis in the chemotaxis of MDSCs, utilizing an in vitro MDSC migration assay. Notably, the deletion of TRIM28 significantly inhibited the migration of MDSCs towards the conditioned medium obtained from CMT-167 cells. Conversely, the introduction of TRIM28 into CMT-167 cells led to an increase in MDSC migration. Importantly, this effect was counteracted when an anti-CXCL1 neutralizing antibody was added to the conditioned medium (Fig. 6A). Furthermore, our analysis of TCGA data revealed a positive correlation between CXCL1 expression and the presence of MDSCs in NSCLC (Fig. 6B).

To validate these findings in a more physiologically relevant setting, we conducted experiments in an autochthonous model of NSCLC, utilizing the *Kras*^*G12D*^; *p53*^*flox/flox*^ (KP) lung adenocarcinoma system, which can be induced through lentiviral delivery of Cre recombinase alone or in combination with TRIM28 (Fig. 6C). Initially, we overexpressed TRIM28 in the KP adenocarcinoma model to investigate its impact on tumor development and MDSC levels. KP mice were infected with lenti-TRIM28-Cre or control lenti-GFP-Cre viruses. In mice infected with lenti-TRIM28-Cre (KP-TRIM28), histological analysis revealed significantly larger tumor sizes and areas in the lungs compared to control KP mice (Fig. 6D). Importantly, TRIM28 expression was substantially upregulated in the lungs of KP-TRIM28 mice (Fig. 6E). Furthermore, the enhanced expression of TRIM28 led to a significant reduction in the survival of KP-TRIM28 mice (Fig. 6G). To further investigate the association between TRIM28 expression and MDSC infiltration, we examined the percentages and numbers of MDSCs infiltrating the tumor-burdened lungs in KP-TRIM28 and KP mice.

Our observations indicated an increase in MDSCs and a decrease in CD8^+^T cell infiltration in the tumor-burdened lungs of KP-TRIM28 mice compared to KP mice (Fig. 6E-F). This was further supported by flow cytometry data, which showed a significant elevation in both the percentages and absolute numbers of MDSCs in the lungs of KP-TRIM28 mice, while the numbers of CD8^+^T cells and CD4^+^T cells were significantly reduced in comparison to KP mice (Fig. 6H-I).

It is widely acknowledged that antigen-presenting cells that capture tumor antigens and then migrate to lymphoid organs, where they activate tumor-specific naïve T cells, leading to their differentiation into effector T cells, play a crucial and central role in anti-tumor immunity. Thus, we assessed adaptive immune responses in the bronchial draining lymph nodes (dLNs). Flow cytometry analysis revealed a significant decrease in the numbers of CD4^+^, CD8^+^, CD4^+^IFNγ^+^ T cells, and CD8^+^IFNγ^+^ T cells in the bronchial dLNs of KP-TRIM28 mice when compared to KP mice. Additionally, there was an increase in the abundance of Treg cells in the dLNs of KP-TRIM28 mice compared to KP mice (Supplementary Fig. 5). These results further underscored the involvement of TRIM28 in reprogramming the tumor microenvironment towards immunosuppression.

To gain a deeper understanding of the different suppressive characteristics of MDSCs in the tumor-burdened lungs of KP-TRIM28 and KP mice, we examined the production and expression of factors associated with the immunosuppressive activity of MDSCs. We observed that MDSCs isolated from the lungs of KP-TRIM28 mice exhibited significantly higher levels of arginase-1 (Arg1), S100A9, and inducible nitric oxide synthase (iNOS) compared to MDSCs from the lungs of KP mice (Fig. 6J). Furthermore, we investigated whether TRIM28 influenced the suppressive function of MDSCs. MDSCs isolated from KP tumors were co-cultured with CD8^+^ T cells in vitro. Remarkably, MDSCs from KP-TRIM28 tumors displayed significantly stronger suppressive activity against the proliferation of CD8^+^T cells when compared to MDSCs from control tumors (Fig. 6K). These findings collectively demonstrated the crucial role of the TRIM28-CXCL1 axis in recruiting MDSCs into the lung cancer microenvironment.

### RIPK1 inhibition sensitizes lung tumors to PD-1 blockade

**Fig. 7:**
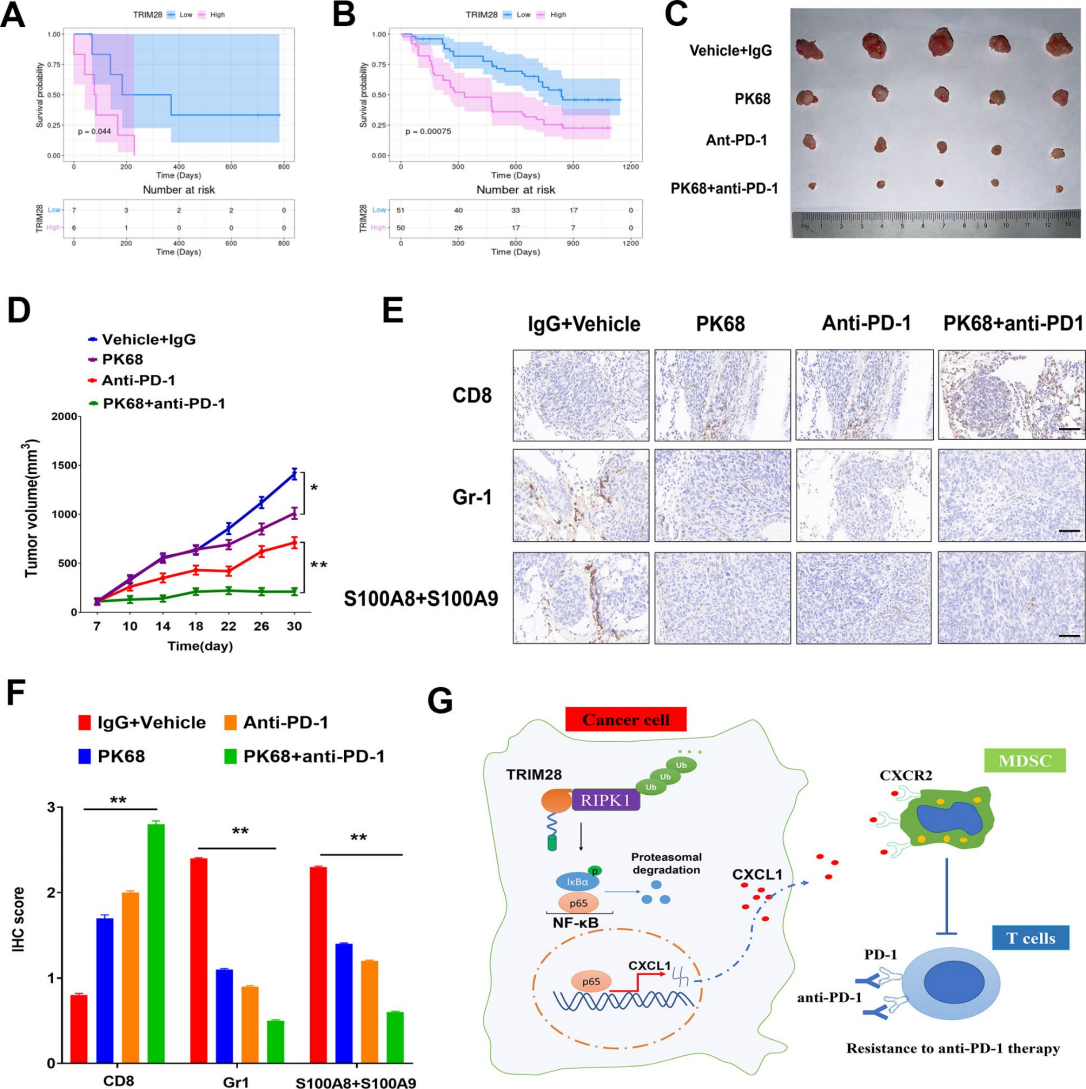
Targeting RIPK1 increases the sensitivity of lung tumors to anti-PD-1 therapy. (**A**) Kaplan-Meier curves predicting survival of LUSC patients receiving anti-PD-1 therapy based on net changes in TRIM28 mRNA levels in the LUSC-GSE93157-anti-PD-1 datasets. (**B**) Kaplan-Meier curves predicting survival of melanoma patients receiving anti-PD-1 therapy based on net changes in TRIM28 mRNA levels in the melanoma-GSE91061-anti-PD-1 datasets. (**C**-**D**) C57BL/6J mice were subcutaneously injected with CMT-167 cells and treated with anti-PD-1, PK68 (RIPK1 inhibitor), PK68 plus anti-PD-1, or isotype control and vehicle. Tumor growth was monitored until the experimental endpoints. Data are shown as mean ± SEM. Tumor growth curves were shown. (**E**-**F**) Representative images of IHC for CD8, Gr-1, and S100A8 + S100A9 in indicated mouse tumors (**E**) and IHC quantification (**F**). The scale bars represent 50 μm. Error bars indicate mean ± SEM. Statistics calculated using one-way ANOVA post hoc Tukey test for multi-group or two-tailed Student’s t-test for two-group comparisons. **p < 0.01. (**G**) High TRIM28 expression is positively correlated with MDSCs infiltration in multiple cohorts of cancer patients. TRIM28 promotes NF-κB activation by regulating K63-linked ubiquitination of RIPK1, leading to increased expression of the cytokine CXCL1, a chemoattractant for MDSCs via the CXCL1-CXCR2 axis. TRIM28-recruited MDSCs antagonize effector CD8^+^T cells in the tumor immune microenvironment of NSCLC, promoting anti-PD-1 resistance

Based on our above findings, we next analyzed the predictive role of TRIM28 in cancer patients undergoing anti-PD-1 therapy. Our analysis revealed that in patients with NSCLC, those with low TRIM28 expression experienced more favorable survival rates and longer survival times in comparison to patients with high TRIM28 expression (Fig. 7A). This relationship between TRIM28 expression and patient response was consistent in the GSE91061 melanoma cohort, where melanoma patients with low TRIM28 expression exhibited improved survival outcomes compared to those with high TRIM28 expression (Fig. 7B).

PK68 is a potent and selective inhibitor of RIPK1 and also highlights its great potential for use in the treatment of cancer metastasis [32]. Given that tumor-associated TRIM28 induces CD8^+^T cell exhaustion through NF-κB signaling, we sought to assess RIPK1 as a potential therapeutic target in combination with immune checkpoint blockade. To explore this, we evaluated the impact of RIPK1 inhibition in a syngeneic mouse model. While the RIPK1 inhibitor PK68 exhibited a modest reduction in tumor growth when administered as a monotherapy, a significant reduction in tumor growth was observed only when PK68 was combined with anti-PD-1 treatment. This reduction in tumor growth became apparent within one week of treatment initiation and was sustained throughout the treatment course (Fig. 7C-D). Immunohistochemical analysis of tumor tissues revealed that treatment with the RIPK1 inhibitor alone enhanced the infiltration of CD8^+^T cells within CMT-167 tumors. However, the PK68/anti-PD-1 combination therapy resulted in a significantly greater infiltration of CD8 + T cells compared to monodrug treatment, a crucial characteristic associated with the success of ICB-based therapy. Furthermore, this combination therapy led to a notable reduction in the infiltration of MDSCs within the tumor microenvironment (Fig. 7E-F). Collectively, our study findings reveal a potential combined therapeutic strategy utilizing a RIPK1 inhibitor and PD-1 blockade to enhance anti-tumor efficacy.

## Discussion

The limited durability of responses to anti-PD-1/PD-L1 immunotherapies in many lung cancer patients underscores the importance of identifying tumor cell-intrinsic and tumor microenvironment resistance mechanisms. Additionally, there is a pressing need to characterize biomarkers for patient selection and to develop rational combinatorial therapeutic strategies [33–35]. Our study demonstrated that TRIM28 exhibits high expression in NSCLC tumor tissues and is positively correlated with the infiltration of MDSCs in the tumor microenvironment. We found that inhibiting TRIM28 in syngeneic mouse models reduced MDSCs in the tumor microenvironment and rendered the tumors more responsive to PD-1 blockade. Further investigations revealed that elevated TRIM28 levels increased the presence of MDSCs within tumors and hindered the response to anti-PD-1 treatment. Mechanistically, TRIM28 promotes the recruitment of MDSCs via chemokine-driven mechanisms facilitated by RIPK1-mediated NF-κB activation. This, in turn, leads to the suppression of CD8^+^T cell activity and contributes to anti-PD-1 resistance. To better replicate the immune tolerance mechanisms observed in human lung cancers, we expanded our analysis to autochthonous tumors in KP mice. By overexpressing TRIM28, these tumors establish an immunosuppressive microenvironment that impedes the antitumor immune response. These findings underscore the significance of tumor TRIM28 expression as a key regulator of immunosuppression within the tumor microenvironment and provide a compelling rationale for targeting this pathway therapeutically.

NF-κB transcription factors have long been known for their central role in orchestrating immune and inflammatory responses [15]. NF-κB can be activated through two distinct pathways: the canonical and the non-canonical pathways. The canonical pathway leads to the activation of NF-kB heterodimers comprising p50 and p65, or p50 and c-Rel, primarily involved in immune activation and cell survival. In contrast, the non-canonical pathway results in the nuclear translocation of p52-RelB heterodimers and is mainly associated with lymphoid organogenesis [14]. Activation of the classical NF-κB pathway in response to inflammatory signals is detrimental to the effectiveness of antitumor immunotherapy. Recent research has highlighted the immunosuppressive role of the classical c-Rel NF-κB subunit, as it contributes to the maintenance of activated Tregs, a subset of tumor-infiltrating Tregs known to inhibit effector CD8^+^T cells [36]. Chemical inhibition of c-Rel function delayed melanoma growth by impairing Treg-mediated immunosuppression and potentiated the effects of anti-PD-1 immunotherapy. Notably, the reduction in tumor volume was much more pronounced when anti-PD-1 therapy was combined with c-Rel inhibition compared to anti-PD-1 treatment alone, underscoring the necessity of inhibiting c-Rel activity for optimal PD-1 inhibitor efficacy [36]. Furthermore, upon TNFα stimulation, the activation of the p65 NF-κB subunit mediates T cell immunosuppression by upregulating PD-L1 expression. Additionally, NF-κB-mediated upregulation of CCL2 promoted the infiltration of tumor-associated macrophages and tumorigenesis in lung cancer [37]. It has been reported that TNF-α activates the NF-κB pathway, leading to the rapid recruitment of TRAF2 and RIPK1 to the membrane TNF receptor, forming complex I, and subsequently undergoing K63-linked ubiquitination, a pivotal event in the activation of the NF-κB pathway [38]. Our results corroborate that TRIM28 physically interacts with RIPK1 and promotes K63-linked ubiquitination of RIPK1, thus sustaining the eventual activation of the NF-κB pathway. These findings establish a crucial connection between TRIM28 expression and the recruitment of MDSCs to tumors, introducing a new pathway through which TRIM28 mediates immunosuppression.

The tumor suppressive function exerted by TRIM28 is closely tied to its control over the expression of pro-tumorigenic chemokines [39]. Elevated levels of CXCL1 in tumors have been shown to play a fundamental role in tumor malignancy and metastasis across various cancer types. CXCL1 functions as a chemokine that attracts MDSCs to the tumor microenvironment by signaling via the G protein-coupled chemokine receptor CXCR2 [30, 40]. CXCR2 is predominantly expressed in myeloid populations such as neutrophils, monocytes, and macrophages. This receptor guides myeloid-derived cells from the bone marrow to CXCL1-overexpressing tumor sites, where they facilitate tumor immune evasion by suppressing the proliferation, activation, and motility of effector T cells and promoting the numerical expansion of Tregs [41, 42]. Consistently, we observed that TRIM28 mediates the migration of MDSCs through the CXCL1-CXCR2 axis. Collectively, our results demonstrate that TRIM28-mediated p65 signaling promotes the recruitment of MDSCs via CXCL1 secretion. Further exploration is warranted to assess the broader impact of TRIM28 on the NF-κB-dependent expression of cytokines and chemokines within the immunosuppressive tumor microenvironment of NSCLC.

It is now well-established that MDSCs contribute to tumor progression by inhibiting immune responses and promoting various aspects of tumor growth, including angiogenesis, tumor cell invasion, and metastasis [43, 44]. A growing body of evidence supports a strong association between the accumulation of MDSCs in peripheral blood and clinical outcomes in cancer patients. A recent meta-analysis involving 442 patients with various solid tumors demonstrated a significant association between peripheral blood MDSCs and poor overall and progression-free survival [45]. MDSCs have also been implicated in resistance to various anti-cancer therapies, including sunitinib, cisplatin, doxorubicin, and melphalan, highlighting their role in promoting resistance to treatment in lung cancer and multiple myeloma. Recent studies have indicated that lower levels of MDSCs are associated with positive clinical responses to immunotherapies such as ipilimumab and PD-1 antibodies [46]. MDSCs-mediated suppression of antitumor T cell activity is a major contributor to the immunosuppression observed within the tumor microenvironment. The tumor microenvironment is a complex milieu housing an array of regulatory lymphocytes interacting directly or indirectly with MDSCs to modulate effector T cell function [47]. Prior studies have shown that coculture with MDSCs results in the impairment of cytotoxic activity of γδ T cells. Furthermore, this impairment might be due to the inhibition of IFN-γ production and degranulation of activated γδ T cells by MDSCs [47]. Notably, the positioning of immune cells within tumors is known to dictate their function. Our results suggest that tumor-expressed TRIM28 spatially separated from MDSC [48]. Therefore, further investigations are needed to determine whether TRIM28 expression could affect the spatial distribution of tumor-associated immune cells. Previous studies have identified various mechanisms through which MDSCs suppress T cells, including the local depletion of nutrients (arginine and cysteine) necessary for T cell activation via Arg1-mediated pathways and the production of nitric oxide (NO) facilitated by iNOS, both of which hinder T cell function. Our findings align with these observations, as we demonstrate that overexpressing TRIM28 in cancer cells leads to the recruitment of functional MDSCs, which subsequently inhibit the function and proliferation of effector T cells in KP tumor-bearing mice, ultimately compromising immune surveillance.

## Conclusions

In conclusion, our study establishes that TRIM28 expression in NSCLC plays a pivotal role in recruiting immunosuppressive MDSCs by activating the RIPK1-p65-CXCL1 axis, leading to the suppression of CD8^+^T cell activity and contributing to anti-PD-1 resistance. The combination of TRIM28 targeting with anti-PD1 therapy demonstrates promising anti-tumor efficacy against NSCLC, highlighting TRIM28 as a potential therapeutic target for this cancer type.

### Electronic supplementary material

Below is the link to the electronic supplementary material.

Supplementary Material 1

Supplementary Material 2

Supplementary Material 3

Supplementary Material 4

Supplementary Material 5

Supplementary Material 6

Supplementary Material 7

Supplementary Material 8

## Data Availability

The data presented in this manuscript are available upon reasonable request from the corresponding author.

## Abbreviations

NSCLC: Non-small cell lung cancer
TRIM: Tripartite motif
ELISA: Enzyme-linked Immunosorbent assay
KP: Kras^LSL−G12D/+^; Tp53^fl/fl^
TIMER2.0: Tumor Immune Estimation Resource 2.0
NF-κB: Nuclear Factor NF-Kappa-B
ICIs: Immune checkpoint inhibitors
TILs: Tumor-infiltrating lymphocytes
OS: Overall survival
MDSCs: Myeloid-derived suppressors cells
RIPK1: Receptor-interacting protein kinase 1
TNF-α: Tumor necrosis factor-α
RT-qPCR: Reverse transcription quantitative PCR
dLNs: Draining lymph nodes
TME: Tumor microenvironment
Arg1: Arginase-1
iNOS: Inducible nitric oxide synthase
