# Supplementary material for: E3 ligase TRIM28 promotes anti-PD-1 resistance in non-small cell lung cancer by enhancing the recruitment of myeloid-derived suppressor cells
Source: J Exp Clin Cancer Res. 2023 Oct 21;42:275. doi: 10.1186/s13046-023-02862-3 (PMC10589970; doi:10.1186/s13046-023-02862-3)
Supplement: Supplementary file 1 — Supplementary Material 1 [file 13046_2023_2862_MOESM1_ESM.docx]

**Supplemental table1. Related to Fig.1A. Tumors abbreviation**

| Abbreviations | Full name |
| --- | --- |
| ACC | Adrenocortical Carcinoma |
| BLCA | Bladder Urothelial Carcinoma |
| BRCA | Breast Invasive Carcinoma |
| CESC | Cervical and Endocervical Cancer |
| CHOL | Cholangiocarcinoma |
| COAD | Colon Adenocarcinoma |
| DLBC | Lymphoid Neoplasm Diffuse Large B-cell Lymphoma |
| ESCA | Esophageal Carcinoma |
| GBM | Glioblastoma Multiforme |
| HNSC | Head and Neck Cancer |
| KICH | Kidney Chromophobe |
| KIRC | Kidney Renal Clear Cell Carcinoma |
| KIRP | Kidney Renal Papillary Cell Carcinoma |
| LAML | Acute Myeloid Leukemia |
| LGG | Lower Grade Glioma |
| LIHC | Liver Hepatocellular Carcinoma |
| LUAD | Lung Adenocarcinoma |
| LUSC | Lung Squamous Cell Carcinoma |
| MESO | Mesothelioma |
| OV | Ovarian Serous Cystadenocarcinoma |
| PAAD | Pancreatic Adenocarcinoma |
| PCPG | Pheochromocytoma and Paraganglioma |
| PRAD | Prostate Adenocarcinoma |
| READ | Rectum Adenocarcinoma |
| SARC | Sarcoma |
| SKCM | Skin Cutaneous Melanoma |
| STAD | Stomach Adenocarcinoma |
| STES | Stomach and Esophageal Carcinoma |
| TGCT | Testicular Germ Cell Tumors |
| THCA | Thyroid Carcinoma |
| THYM | Thymoma |
| UCEC | Uterine Corpus Endometrial Carcinoma |
| UCS | Uterine Carcinosarcoma |
| UVM | Uveal Melanoma |
