# Supplementary material for: E3 ligase TRIM28 promotes anti-PD-1 resistance in non-small cell lung cancer by enhancing the recruitment of myeloid-derived suppressor cells
Source: J Exp Clin Cancer Res. 2023 Oct 21;42:275. doi: 10.1186/s13046-023-02862-3 (PMC10589970; doi:10.1186/s13046-023-02862-3)
Supplement: Supplementary file 2 — Supplementary Material 2 [file 13046_2023_2862_MOESM2_ESM.docx]

**Supplementary table 2: Analysis of association between TRIM28 expression and clinicopathological parameters in lung cancer**

|  |  | TRIM28 expression | |  |
| --- | --- | --- | --- | --- |
| Characteristics | **N** | **Low** | **High** | ***P* value** |
| Age |  |  |  | 0.221 |
| ≤ 60 | 37 | 12 | 25 |  |
| > 60 | 53 | 11 | 42 |  |
| Gender |  |  |  | 0.473 |
| Male | 49 | 14 | 35 |  |
| Female | 41 | 9 | 32 |  |
| Differentiation |  |  |  | 0.124 |
| Well | 20 | 8 | 12 |  |
| Moderate | 52 | 13 | 39 |  |
| Poor | 18 | 2 | 16 |  |
| Tumor size (T) |  |  |  | <0.001 |
| T1-T2 | 37 | 19 | 18 |  |
| T3-T4 | 53 | 4 | 49 |  |
| Lymph node metastasis |  |  |  | 0.039 |
| N0-N1 | 42 | 15 | 27 |  |
| N2-N3 | 48 | 8 | 40 |  |
| Distant metastasis (M) |  |  |  | 0.039 |
| Negative (M0) | 73 | 22 | 51 |  |
| Positive (M1) | 17 | 1 | 16 |  |
| Tumor stage |  |  |  | <0.001 |
| Ⅰ-Ⅱ | 29 | 18 | 11 |  |
| Ⅲ-Ⅳ | 51 | 5 | 46 |  |

**P* values <0.05 were considered statistically significant (chi-square test for categorical variables).
