# Supplementary figures and images for: E3 ligase TRIM28 promotes anti-PD-1 resistance in non-small cell lung cancer by enhancing the recruitment of myeloid-derived suppressor cells
Source: J Exp Clin Cancer Res. 2023 Oct 21;42:275. doi: 10.1186/s13046-023-02862-3 (PMC10589970; doi:10.1186/s13046-023-02862-3)

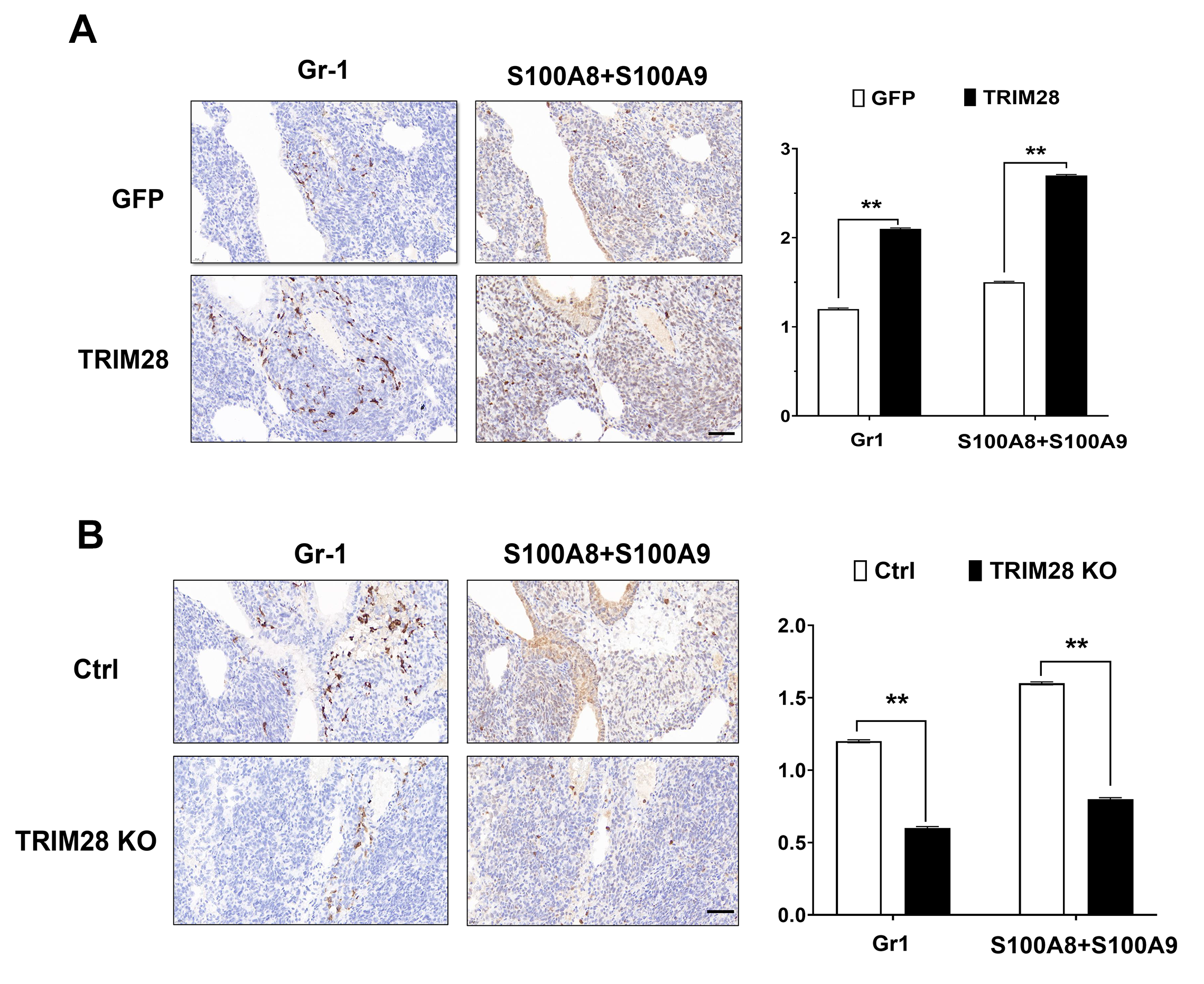

Supplement: Supplementary file 3 — Supplementary Material 3 [file 13046_2023_2862_MOESM3_ESM.png]

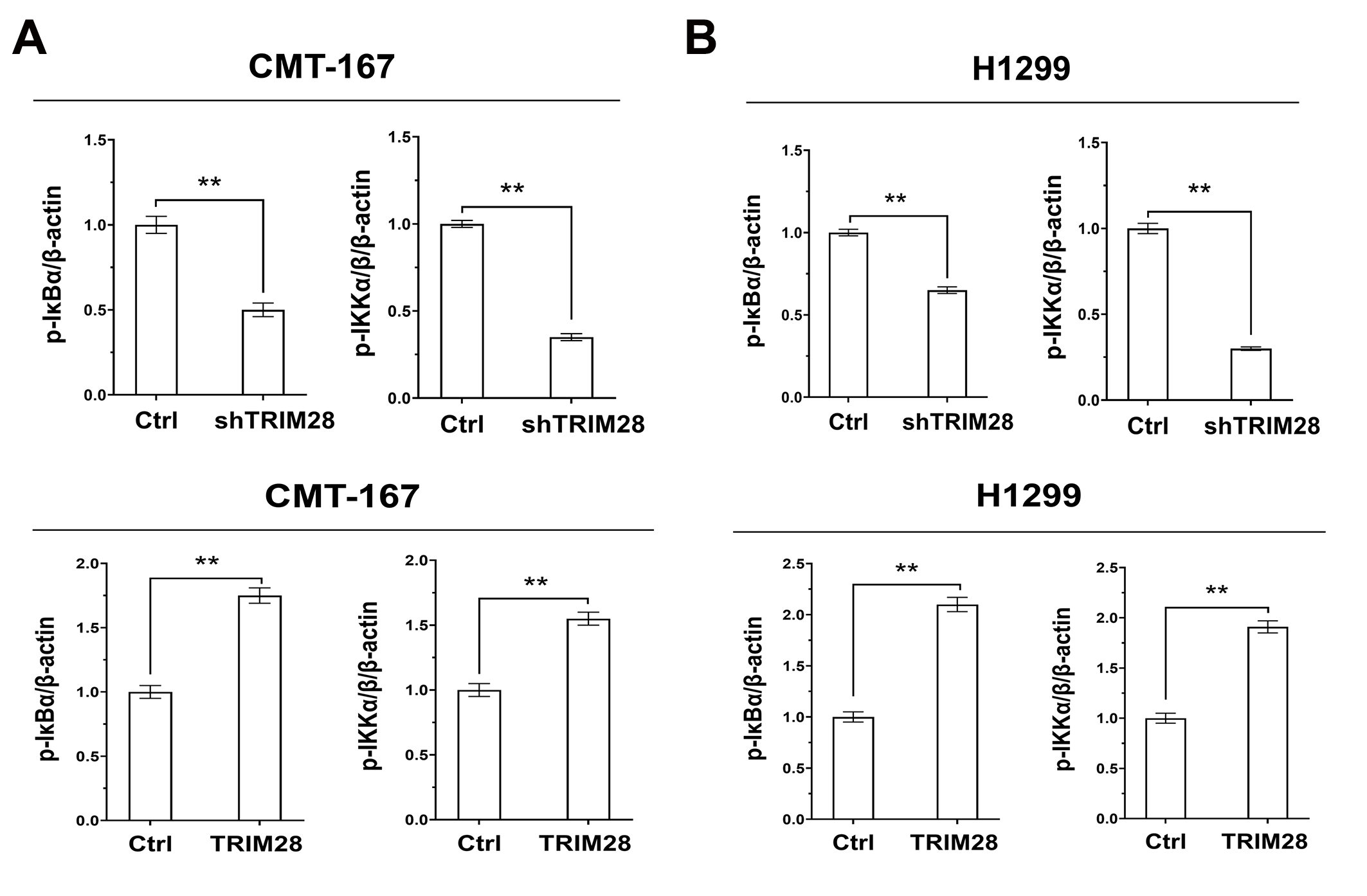

Supplement: Supplementary file 4 — Supplementary Material 4 [file 13046_2023_2862_MOESM4_ESM.png]

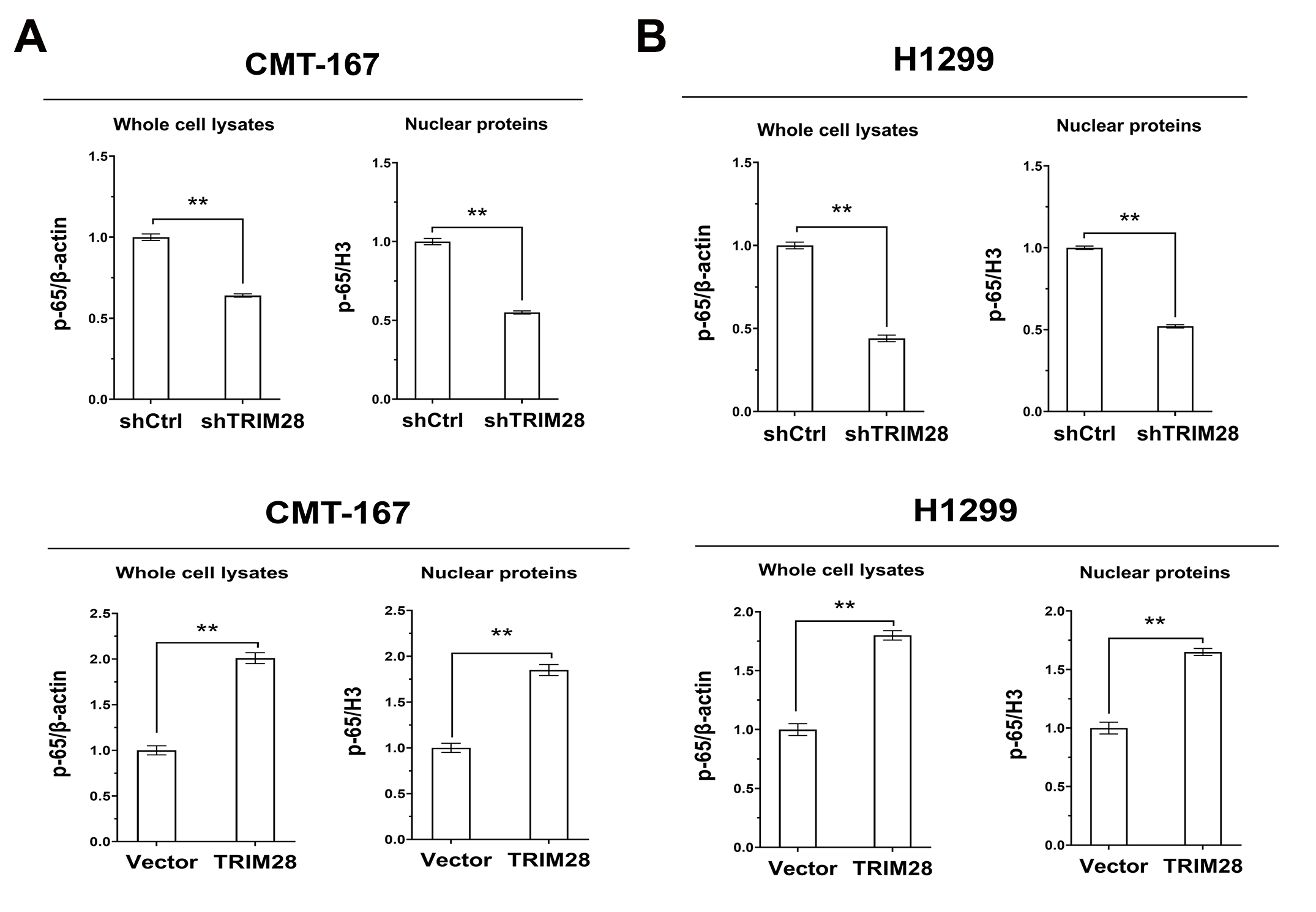

Supplement: Supplementary file 5 — Supplementary Material 5 [file 13046_2023_2862_MOESM5_ESM.png]

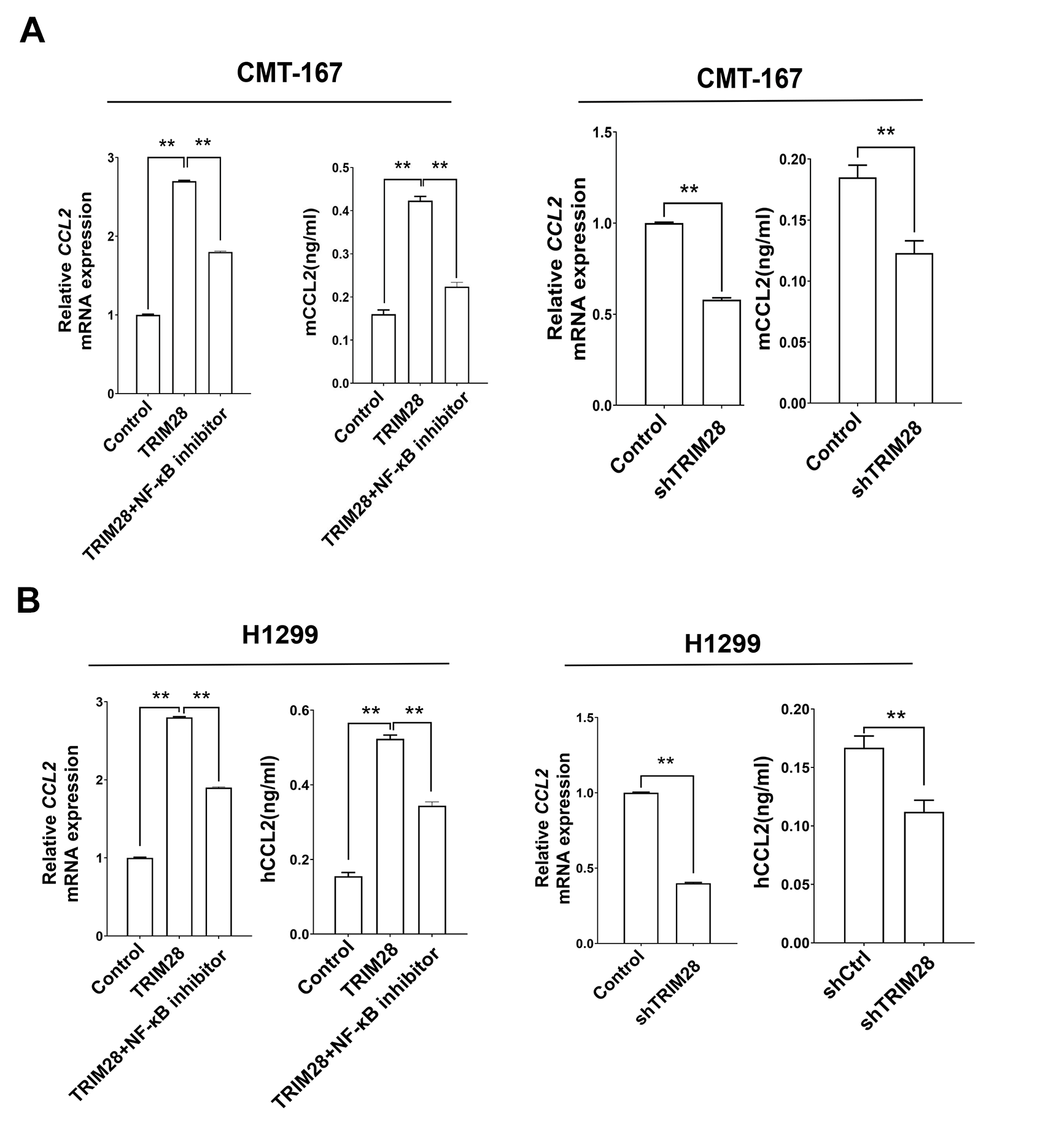

Supplement: Supplementary file 6 — Supplementary Material 6 [file 13046_2023_2862_MOESM6_ESM.png]

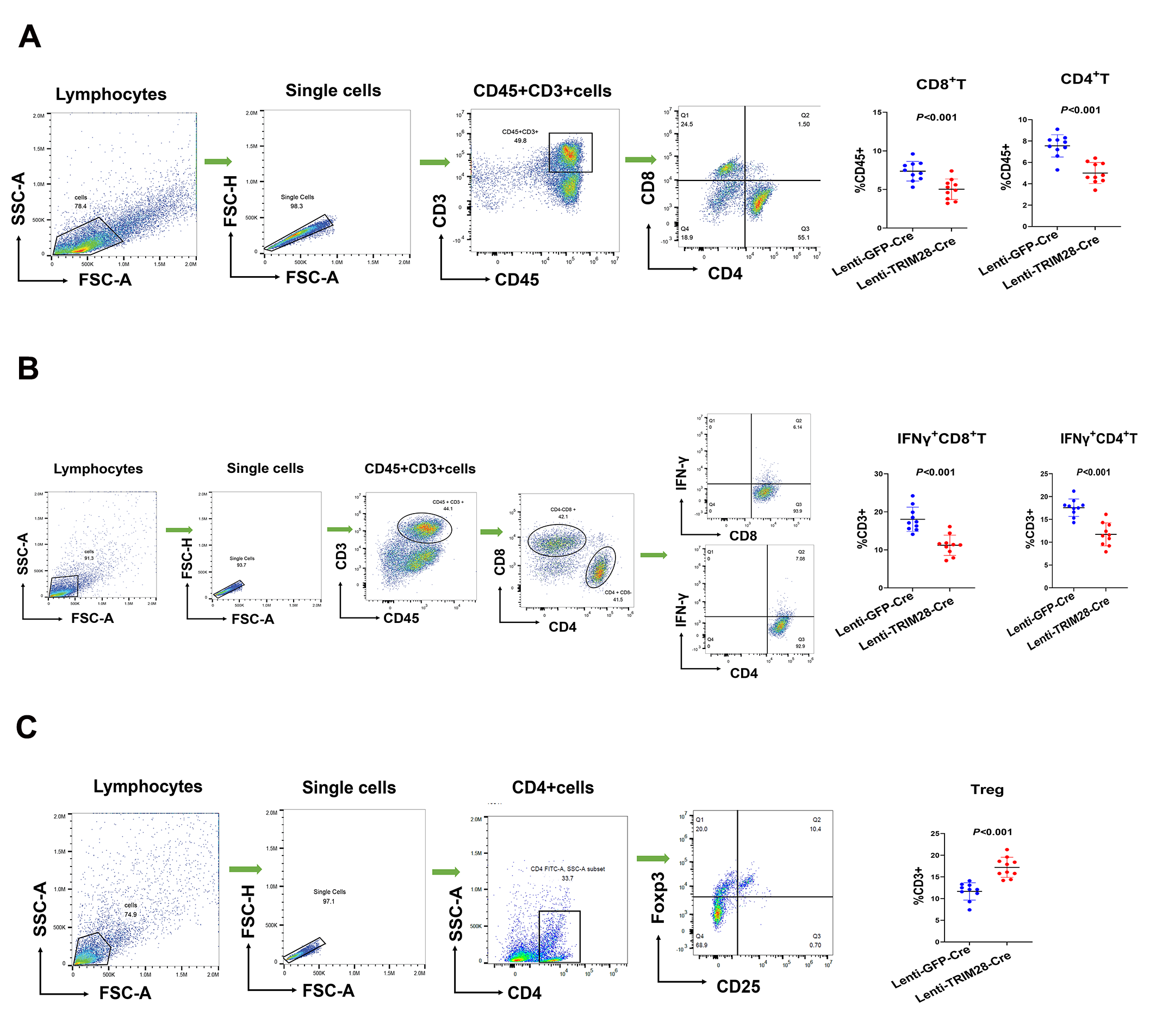

Supplement: Supplementary file 7 — Supplementary Material 7 [file 13046_2023_2862_MOESM7_ESM.png]
