# Supplementary material for: E3 ligase TRIM28 promotes anti-PD-1 resistance in non-small cell lung cancer by enhancing the recruitment of myeloid-derived suppressor cells
Source: J Exp Clin Cancer Res. 2023 Oct 21;42:275. doi: 10.1186/s13046-023-02862-3 (PMC10589970; doi:10.1186/s13046-023-02862-3)
Supplement: Supplementary file 8 — Supplementary Material 8 [file 13046_2023_2862_MOESM8_ESM.docx]

**Figure S1 Related to Figure 2. TRIM28 promotes MDSCs migration to the tumor microenvironment in syngeneic mice.**

(A) Immunohistochemistry (IHC) for Gr-1 and S100A8+S100A9 in subcutaneous tumor from CMT-167 cells infected with lenti-GFP or lenti-TRIM28 in C57BL/J mice. IHC quantification of intra-tumoral MDSC cells (Gr-1 and S100A8+S100A9). The scale bar represents 200μm.

(B) IHC for Gr-1 and S100A8+S100A9 in subcutaneous xenografts from CMT-167 or CMT-167-TRIM28 KO cells and IHC quantification of Gr-1 and S100A8+S100A9. The scale bar represents 200μm. Statistics calculated using two-tailed unpaired t tests, ***p* < 0.01.

**Figure S2** **Related to Figure 4. The expression of TRIM28 is positively correlated with NF-kB signaling in lung cancer cells.**

(A) p-IκBα and p-IKKα/β protein levels were quantified by using densitometry and normalized to β-actin and are shown as fold changes compared to the control in TRIM28-knockdown and TRIM28-overexpressed CMT-167 cells.

(B) p-IκBα and p-IKKα/β protein levels were quantified by using densitometry and normalized to β-actin and are shown as fold changes compared to the control in TRIM28-knockdown and TRIM28-overexpressed H1299 cells. Quantification of western blotting were performed with the Image J software. Statistics calculated using two-tailed unpaired t tests, ***p* < 0.01.

**Figure S3 Related to Figure 5. TRIM28 promotes NF-kB signaling in lung cancer through promoting p65 nucleus translation.**

(A) p-65 protein levels were quantified by using densitometry and normalized to β-actin or H3 and are shown as fold changes compared to the control in TRIM28-knockdown and TRIM28-overexpressed CMT-167 cells.

(B) p-65 protein levels were quantified by using densitometry and normalized to β-actin or H3 and are shown as fold changes compared to the control in TRIM28-knockdown and TRIM28-overexpressed H1299 cells. Quantification of western blotting were performed with the Image J software. Statistics calculated using two-tailed unpaired t tests, ***p* < 0.01.

**Figure S4 Related to Figure 5.** **TRIM28 promotes CCL2** **expression via NF-kB signaling pathway.**

(A-B) RT-qPCR determination of CCL2 mRNA expression in TRIM28-knockdown and TRIM28-overexpressed CMT-167 or H1299 cell, treated with or without NF-κB inhibitor (BAY11-7085) at 10μM for 24h. ELISA validation of levels of CCL2 in cell culture supernatants from CMT-167 or H1299 cell culture. Statistics calculated using one-way ANOVA post hoc Tukey test for multi-group or two-tailed student’s t-test for two-group comparisons. ***p* < 0.01.

**Figure S5. Related to Figure 6. Tumors with TRIM28 overexpression impairs the activation of antitumor immune responses.**

Flow cytometry sample gating strategy for live T cell populations in bronchial dLNs of KP and KP-TRIM28 mice.

(A) Gating strategies to identify CD4^+^ or CD8^+^ T cells in the bronchial dLNs of KP and KP-TRIM28 mice. Right: Quantification of percentage of total CD4^+^T and CD8^+^T cells for each individual sample.

(B) Gating strategies to identify CD4^+^IFN-γ^+^T and CD8^+^ IFN-γ^+^T cells in the bronchial dLNs of KP and KP-TRIM28 mice. Right: Quantification of percentage of CD4^+^IFN-γ^+^T and CD8^+^IFN-γ^+^T cells for each individual sample.

(C) Representative flow cytometry plots for gating strategy of Treg cells. Right: Quantification of percentage of Treg cells for each individual sample. Statistics calculated using two-tailed unpaired t tests, ***p* < 0.01.
